# Integration of Bone Remodeling and Damage Accumulation: A Preliminary Computational Framework for Physiological and Pathological Responses

**DOI:** 10.1007/s10441-026-09522-x

**Published:** 2026-05-04

**Authors:** Diego A. Garzón-Alvarado, Carlos A. Duque-Daza, Dorian L. Linero, Boumediene Nedjar, Abdelghani May, Estevam Barbosa Las Casas, Salah Ramtani

**Affiliations:** 1 Biotechnology Institute, Universidad Nacional de Colombia, Bogotá, Colombia; 2 GNUM, Universidad Nacional de Colombia, Bogotá, Colombia; 3 Laboratoire CB3S, equipe BEST, CNRS (UMR 7244), Université Sorbonne Paris Nord, Villetaneuse, France; 4 Université d’Evry, Paris-Saclay, LMEE, 91020 Evry, France; 5 Ecole Mil Polytech, Mech Syst Design Lab, BP 17, 16214 Algiers, Algeria; 6 Departamento de Engenharia de Estruturas, Escola de Engenharia, Universidade Federal de Minas Gerais – UFMG - Belo Horizonte (MG), Belo Horizonte, Brasil; 7 Civil and Agricultural Engineering Department, Universidad Nacional de Colombia, Bogotá, Colombia

**Keywords:** Bone remodeling, Komarova’s model, Osteoclasts, Osteoblasts, Accumulated damage, Fatigue, Personalized medicine

## Abstract

This study presents a computational model that integrates bone remodeling dynamics with damage accumulation, focusing on both physiological and pathological conditions. Building upon Komarova’s classical model of osteoclast and osteoblast interactions, this work introduces fatigue-induced damage using a stress-life (S-N) approach. By simulating bone responses under sinusoidal and random mechanical loads, the model captures the cyclical nature of bone turnover. The results show that under normal physiological conditions, bone is able to repair microdamage and maintain structural integrity. However, in pathological scenarios such as osteoporosis and tumors, the remodeling cycle is disrupted, leading to an increase in damage accumulation and eventual structural failure. Through numerical simulations, the study also demonstrates the significant impact of fatigue on bone health, showing that repetitive mechanical loads, even below critical stress levels, can result in bone degradation over time. By capturing the accumulation of microdamage and its repair, the model offers potential applications in personalized medicine to assess fracture risks in varying stress and health scenarios. This approach provides a framework for understanding how different stress patterns contribute to bone damage and offers insights into the progression of bone diseases. The model could be extended using metabolic and age-related characteristics and serves as a potential tool for personalized medicine, helping to predict bone failure risks in individuals when they are submitted to repetitive mechanical loads.

## Introduction

Maintaining healthy bone structure is essential for the Earth life because it supports daily movements and physiological functions in both humans and animals through the musculoskeletal system. One of the most important processes within bone metabolism is bone turnover. This process, essential for long-term bone maintenance, occurs via a well-regulated cycle known as bone remodeling. It is regulated by specialized cells, which are organized as a Basic Multicellular Unit (BMU), consisting primarily of osteoclasts and osteoblasts, which degrade or resorb the bone matrix. Bone remodeling takes place continually at random, discrete locations in the skeleton, with osteoclasts initiating bone breakdown and osteoblasts rebuilding the structure afterward (Brand 2010; Martin and Ng 1994; Günther and Schinke 2000; Michael Parfitt 1994).

Historically, the work of pioneers like Wolff (Wolff 1873), Culmann and von Meyer Von Meyer (1867), and Frost Tyrovola (2015) has greatly advanced our understanding of the interplay between bone structure, mechanical forces, and the process of bone remodeling. More recently, Komarova’s research (2003), has become a classical contribution, particularly in illustrating the interactions between osteoclasts and osteoblasts and their influence on bone mass apposition. Komarova’s work has proven crucial for exploring the biological and mechanical aspects of bone behavior. The foundational insights provided by these researchers have allowed further advancements in the field, including the development of mathematical models and computational simulations that give us a deeper understanding of the complex dynamics of bone adaptation and remodeling.

Many studies have built upon Komarova’s work (2003) to highlight other essential aspects of bone remodeling mechanics. An extension of the previous model was presented Bruce P Ayati et al. (2010), where the interactions between osteoclasts and osteoblasts were analyzed under both stable and unstable oscillatory conditions. In the normal scenario, the interactions remained stable, while in the presence of myeloma development, the system became unstable. The study revealed that during myeloma, osteoclast activity increases, while osteoblast activity decreases, leading to a net loss of bone mass as the tumor progresses. In addition, Bonfoh et al. (2011) also utilized the differential equations developed by Komarova et al. (2003) to explain how various factors respond to stimuli, ultimately affecting bone density. Komarova’s model has also been used to extend its zero-dimensional results into two-dimensional domains, as demonstrated by Graham et al. (2012). In their study, the Level Set Method was employed to simulate complex geometries and the interfaces between bone and bone marrow, allowing for an in-depth analysis of the consequences of bone remodeling. This model provided computational representations of both in vivo and in vitro conditions, offering a clearer picture of how bone remodeling processes unfold in different biological environments. Similarly, Hambli (2014) created a finite element (FE) model that describes how bone behaves under fatigue caused by accumulated damage. In that model, Hambli introduced a damage-strain function that regulated the autocrine and paracrine levels of the BMU, based on Komarova’s model. He applied this model to the proximal femur, integrating BMU activity with linear elasticity and damage mechanisms. Under external mechanical loads, the model generated bone density patterns that aligned with previous models and clinical observations.

In our research group, we have been working to integrate Komarova’s bone remodeling model with a cellular population framework that can predict disease progression. This approach includes factors like tissue damage from conditions such as osteoporosis and cancer, expanding the original idea of Komarova’s equations (Ramtani et al. 2023a; Sánchez et al. 2024; Garzón-Alvarado et al. 2024). More recently, we published a study illustrating how Komarova’s model can map the spatial dynamics of bone remodeling, specifically showing how different cellular populations within the extended BMU contribute to remodeling when subjected to external forces (Garzón-Alvarado et al. 2024).

Unlike previous models that primarily focus on the static interactions between bone cells and their immediate environment, our approach introduces a dynamic perspective by incorporating fatigue damage directly into Komarova’s BMU model. This novel integration allows us to investigate how repetitive mechanical loads, even below critical stress levels, can lead to gradual bone degradation over time, providing a more comprehensive understanding of bone failure. We employ a theoretical damage accumulation model based on Minner’s rule (Shigley and Budynas 2019), but our approach offers something new: a time-dependent view where damage is recalculated along time. Unlike earlier models, ours shows how remodeling, specifically the action of osteoclasts removing damaged tissue and osteoblasts generating new bone, influences damage over time. The model provides a dynamic picture of how bone health is preserved, with cycles of damage accumulation and removal aligning with the natural remodeling process.

The aim of this work is to integrate a damage model with Komarova’s BMU model to better understand how damage accumulates over time and how it is removed through the remodeling process. Additionally, the damage model considers the daily load cycles of an average person, making it a potentially valuable tool for individuals to track their physical activity and assess whether their bone tissue is at risk of failure due to accumulated damage, which could lead to fractures. The main contribution of this paper lies in presenting an advanced computational model that integrates the dynamics of bone remodeling with the accumulation of damage due to mechanical fatigue. This model builds on Komarova’s classic model of osteoclast-osteoblast interactions, adding a focus on the effects of fatigue damage under different loading conditions, including sinusoidal and random mechanical loads. Through this integration, the model captures both physiological and pathological responses, simulating the cyclical process of bone renewal and demonstrating how different stress patterns influence bone health and potential failure. Additionally, this model is especially notable for its ability to simulate real-life scenarios, showing that under normal conditions, bone can repair microdamage to maintain structural integrity. However, under pathological conditions (like osteoporosis or tumors), the balance in the remodeling cycle shifts, increasing damage accumulation and raising fracture risk. Thus, the model has potential applications in personalized medicine, enabling predictions about bone failure risks based on individual stress patterns and metabolic factors.

Users can input their activity along time or use average daily activity levels. For our simulations, we implemented cycles of rest (no load), exercise (high mechanical loads), and other activities (intermediate loads). These loads were represented as sinusoidal, random, or step functions. The model successfully demonstrates damage removal and can simulate BMU behavior. Conversely, damage and dysfunction in BMU activity can lead to failure loads, resulting in fractures. Moreover, we have developed a model that can distinguish between fracture, microfracture, and safe conditions. The model also allows adjustments to the stress-fatigue curve to simulate pathological conditions, providing further insights into the effects of disease on bone health.

## Material and Methods

In this work, we applied Komarova’s model, which is based on two nonlinear differential equations. These equations are driven by biochemical signals known as paracrine and autocrine factors (Eq. 1) (Svetlana et al. 2003). The model also incorporates an equation that tracks bone formation and resorption, allowing us to estimate changes in bone mass over time, hence, we have:

$$\begin{aligned} {\left\{ \begin{array}{ll} & \frac{dx_1}{dt}={\alpha }_1 x^{g_{11}}_1x^{g_{21}}_2-\beta _1x_1 \qquad \qquad \qquad \qquad \qquad \qquad \qquad \text {(1a)}\\ & \frac{dx_2}{dt}=\alpha _2 x^{g_{12}}_1x^{g_{22}}_2-\beta _2 x_2\qquad \qquad \qquad \qquad \qquad \qquad \qquad \text {(1b)}\\ & \frac{dz}{dt}={-k}_1y_1+k_2y_2\qquad \qquad \qquad \qquad \qquad \qquad \qquad \qquad \text {(1c)} \mathrm {\ } \end{array}\right. } \end{aligned}$$

In this model, $x_1$ represents osteoclasts and $x_2$ represents osteoblasts, with $g_{ij}$ capturing the influence of one cell type on the other through paracrine signaling (from *i*-th cell to *j*-th one). The variable *z* reflects the percentage of bone mass, adjusted based on how much the current levels of osteoclasts or osteoblasts deviate from their steady-state values, $\overline{x_i}$. Specifically, this deviation is expressed as ${2y}_i = \left( x_i - \overline{x_i}\right) + \mid \left( x_i - \overline{x_i}\right) \mid$.

In this article, we build upon the Komarova models previously developed in Ramtani et al. (2023a, 2023c, 2023b), where extended models were introduced to describe and simulate various pathological behaviors of Bone Multicellular Units (BMUs). As discussed in the cited works, these models explore scenarios such as bone tumors, osteoporosis, and other diseases characterized by abnormal bone mass reduction. These pathologies arise due to the irregular behavior of cells, such as a decrease in osteoblast activity or an excessive increase in osteoclast function.

### S-N Curve in Bone Tissue: An Approximation and Hypothetical Curve

In mechanical design engineering, particularly in machine design, it’s well-known that components can fail due to repeated loading, even if the applied stresses are below the material’s ultimate strength and often well below its yield strength (Shigley and Budynas 2019). This type of failure in bone tissue is often referred to as fatigue fracture. Essentially, the material wears down over time as it’s repeatedly stressed, ultimately leading to failure. Fatigue failure is typically evaluated using stress versus life (S-N) curves.

There are three main methods to assess fatigue life: the stress-life method, the strain-life method, and linear elastic fracture mechanics (Shigley and Budynas 2019; Hambli 2014). Each of these methods attempts to predict how long a material will last, in terms of the number of cycles before failure, at specific load levels. Although the strain-life method is more accurate (Shigley and Budynas 2019; Hambli 2014), the stress-life method is far more commonly used due to its simplicity and wide range of applications (Shigley and Budynas 2019). In this context, we will apply the stress-life method to predict fatigue failure in bone.

**Fig. 1:**
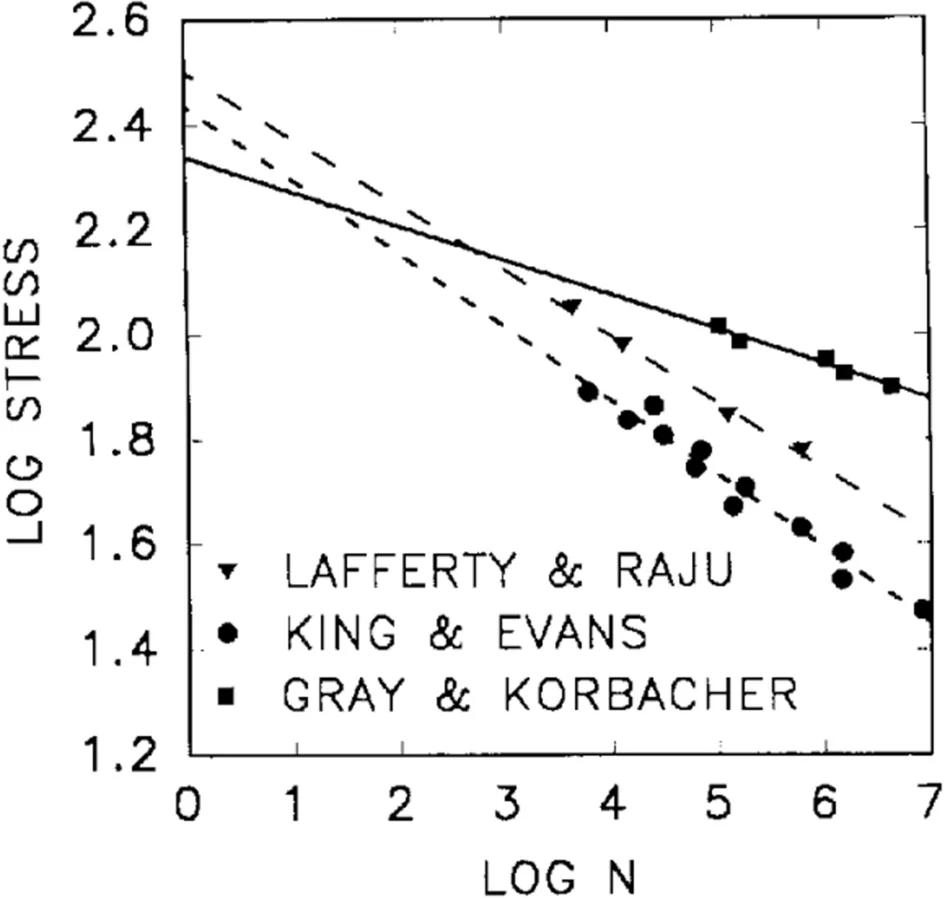
The S-N diagrams from tests done by Gray and Korbacher (1974) on a cow femur in compression, King and Evans (1967) on an embalmed human femur as reversed cantilevers, and Lafferty and Raju (1979) on a bovine femur as rotating cantilevers show the fatigue curves. Stress was measured in megapascals before logarithmic transformation. Axes are base-10 logarithmic: the abscissa is $\log _{10} N$ (cycles to failure) and the ordinate is $\log _{10} S$ (MPa). Thus, $\log _{10} N = 7$ corresponds to $N = 10^{7}$ cycles (not 7 cycles). The decrease from $\log _{10} S = 2.4$ to 1.6 corresponds approximately to $S \approx 250 \rightarrow 40$ MPa, consistent with the expected reduction in allowable fatigue stress with increasing life ($S_f = S_{ut} N^{\,b}$ with *b<0*). Figure taken from Martin (1992).

Another approach to predicting bone failure is through fracture mechanics, which assumes a pre-existing crack and calculates how it grows under stress cut out Shigley and Budynas (2019). For more details on bone damage due to fracture, see the classical reference (Martin 1992) and also recent important works (Malekipour et al. 2022; Meng et al. 2023; Kolken et al. 2022; Meng et al. 2021; Frank et al. 2020; Vila Pouca et al. 2020; Lin et al. 2020; Mouss et al. 2020). For instance, it is shown stress-life experiments were performed on cow bones and human cadaver bones, and the original data is summarized in the classic work and shown in Fig. 1 taken from (Martin 1992). In this work, we will use a stress-life (S-N) curve available in the literature, as mentioned above. This does not imply that there are no newer or more advanced S-N models; rather, we will utilize a well-established and accepted curve, such as the one presented in King and Evans (1967), for the purpose of demonstrating the integration model developed here.

In the study by King and Evans King and Evans (1967), the fatigue stress curve for bone is reported (see Fig. 1). From this graph, the following equation can be obtained:

2 $$\begin{aligned} \log S_f = \log S_{ut} + b \log N \end{aligned}$$

where $S_f$ is the fatigue limit at a given number of cycles *N*, $S_{ut}$ is the ultimate strength, and *b* is the slope of the curve.

This relationship can also be expressed as Shigley and Budynas (2019):

3 $$\begin{aligned} S_f = S_{ut} N^b \end{aligned}$$

Using data from King and Evans King and Evans (1967), the equation takes the following form:

4 $$\begin{aligned} S_f = 281.838 N^{-0.143} \end{aligned}$$

It is important to note that this is an estimated value, serving primarily as an approximation to illustrate our approach to modeling bone damage using the Komarova model. Future research on developing stress-life curves should build upon the foundation laid by these and other researchers (Malekipour et al. 2022; Meng et al. 2023; Kolken et al. 2022; Meng et al. 2021; Frank et al. 2020; Vila Pouca et al. 2020; Lin et al. 2020; Mouss et al. 2020).

In addition, as Shigley (Shigley and Budynas 2019) highlights, there are various factors that can alter the fatigue strength limit. Fatigue equations are typically developed under laboratory conditions, which may differ from real-world scenarios. Therefore, in our approach, we have introduced correction factors for fatigue strength following Marin’s method (Martin 1992), as follows:

5 $$\begin{aligned} S_{ut}^* = k_a k_b k_c S_{ut} \end{aligned}$$

Here, $0< k_a, k_b, k_c < 1$ are factors that reduce fatigue strength, and we can hypothetize that those factors are due to variables such as metabolic syndrome, bone diseases and aging.

**Fig. 2:**
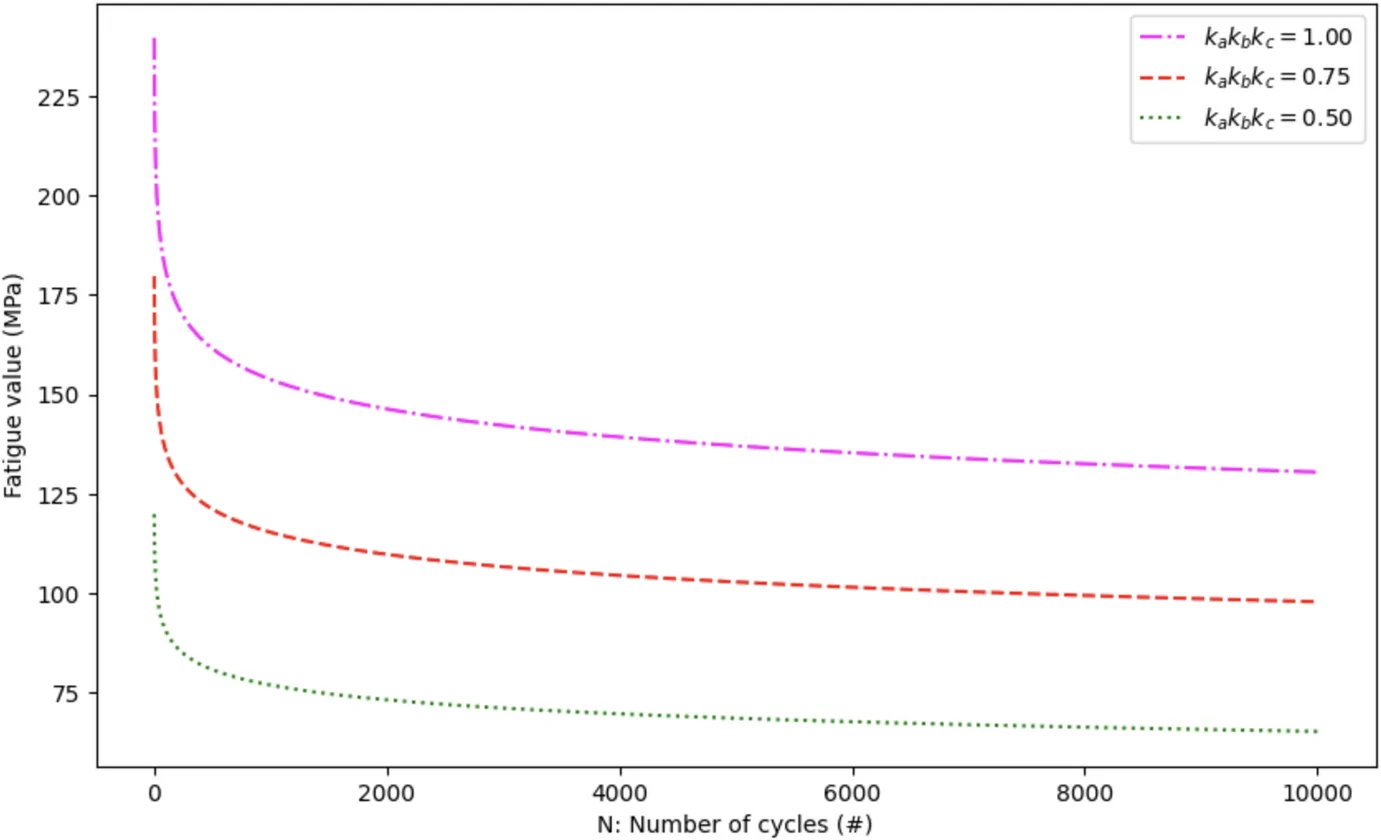
Fatigue strength versus cycles on a *linear* N-axis (same S–N relation as Fig. 1). The shorter range (up to $10^{4}$ cycles) and linear scaling produce a visually milder decline; when replotted on log–log axes or extended to $10^{7}$ cycles, the curve is consistent with Fig. 1.

### Failure Criteria and Variable Stress

Daily activities can subject bones to varying loads, including mean stresses, alternating stresses, and unexpected load peaks that must be taken into account. It is therefore essential to understand the mean stress, amplitude, and alternating components in order to establish appropriate failure criteria, which can predict the behavior of bone tissue under fluctuating loads that may lead to fatigue. Geometric "failure loci" have been developed (a method commonly used in mechanical components) to assess areas with high probability to failure (Shigley and Budynas 2019). Among these criteria, the Gerber, Goodman, and Soderberg methods are widely accepted (Shigley and Budynas 2019). For illustrative purposes in this study, we will use the Gerber criterion, expressed as:

6 $$\begin{aligned} \frac{\sigma _a}{S_f} + \left( \frac{\sigma _m}{S_\text {ut}}\right) ^2 = 1 \end{aligned}$$

where $\sigma _a$, $\sigma _m$, $S_f$, and $S_\text {ut}$ represent the alternating stress, mean stress, fatigue limit (this last given by Eq. 3), and ultimate tensile strength (from Eqs. 3 and 4), respectively. The Gerber criterion states that failure will occur when the ratio of these values reaches one, as indicated by the equation. This criterion is particularly useful for understanding how alternating and mean stresses affect the fatigue limit, as shown below:

7 $$\begin{aligned} S_f = \frac{\sigma _a}{1 - \left( \frac{\sigma _m}{S_\text {ut}}\right) ^2} \end{aligned}$$

This indicates that if the mean stress $\sigma _m$ is much lower than $S_\text {ut}$, the fatigue strength will be largely determined by the alternating stress $\sigma _a$ (deviating from the mean). Conversely, if the mean stress is also high, the fraction becomes larger, increasing the risk of fatigue failure. This concept can be illustrated by considering an overweight individual (with high mean stress $\sigma _m$) starting an impact-heavy exercise regimen (resulting in a high alternating stress $\sigma _a$). In this case, the risk of fatigue fracture is higher for the elevated mean stress, compared to a person with lower body weight.

Although the Gerber mean-stress correction was originally proposed for ductile metals, it is employed here as a phenomenological and numerically stable mean-stress adjustment within a preliminary coupled damage–remodeling framework. We acknowledge that Gerber may be non-conservative for brittle or quasi-brittle behavior under tensile mean stress and may therefore underestimate fatigue risk in that regime. For this reason, the present results should be interpreted within the model’s scope, and the proposed formulation is modular: Gerber can be directly replaced by more conservative relations (e.g., modified Goodman or Soderberg-type corrections) or bone-specific calibrations without altering the overall coupling strategy. It can be included in the future work of our research group.

### Damage Accumulation

Incorporating Komarova’s model, along with the equations described below, we integrate a mechanical damage model to account for the accumulation of damage. Each cycle begins with the removal of damaged tissue by osteoclasts, which erode and destroy old tissue, making way for new tissue laid down by osteoblasts Ramtani et al. (2023a, c). This process results in a daily cycle of stress accumulation, following Palmgren-Miner’s rule (Shigley and Budynas 2019).

In each time step, a load cycle is applied, which contributes to damage accumulation. For a given stress levels given by $\sigma _a$ and $\sigma _m$, the number of cycles needed to cause failure (fracture) in the bone is denoted as $N_i$, which can be obtained from 3, as follows

8 $$\begin{aligned} N = \left( \frac{S_f}{S_{ut}}\right) ^\frac{1}{b} \end{aligned}$$

If a cycle is performed at a stress level lower than *N*, no immediate failure occurs, but cycles can accumulate over time (with mechanical loads of different intensities). According to Miner’s rule, the accumulated damage is calculated as follows:

9 $$\begin{aligned} D_d = \sum _{i=1}^{\text {total cycles}} \frac{n_i}{N_i} \end{aligned}$$

where $N_i$ represents the number of cycles to failure due to accumulated damage.

### Modification of Komarova’s Model

It is important to note that this study takes into account the external loading cycles to which bone tissue is subjected. While the reduction in ultimate strength is considered through Marin’s equation Shigley and Budynas (2019), accounting for pathological factors, alterations in the bone remodeling cycle may also occur, which cannot be directly incorporated (though they can be explored through the use of a time-related factor). Therefore, disturbances in the bone remodeling cycle can be addressed using models such as those presented by Ramtani et al. (2023a, 2023c, 2023b), where modifications of Komarova’s equation were employed, introducing a pathophysiological modulation parameter *P* (dimensionless), which represents non-mechanical biochemical influences on BMU cell activity and is used to construct pathological remodeling scenarios by modulating the kinetic terms of Komarova’s model.. This parameter can lead to changes in the remodeling cycle, either by increasing or decreasing its period and/or altering the bone deposition properties.

Following the previous work of Ramtani et al. (2023b), the following modifications to Komarova’s model will be implemented in this study.

#### Tumor Affecting the Activity of Cell Production or Removal

Building on the work of Ramtani et al. (2023a, 2023b, 2023c), we introduced a new parameter *P* leading to the following modified versions of Eq. (1) which become:

$$\begin{aligned} {\left\{ \begin{array}{ll} & \frac{dx_1}{dt}=\widehat{{\alpha }_1\left( P\right) }x^{g_{11}}_1x^{g_{21}}_2-\widehat{{\beta }_1(P)}x_1 \quad \qquad \qquad \qquad \qquad \qquad \text {(10a)}\\ & \frac{dx_2}{dt}=\widehat{{\alpha }_2\left( P\right) }x^{g_{12}}_1x^{g_{22}}_2-\widehat{{\beta }_2(P)}x_2 \quad \qquad \qquad \qquad \qquad \qquad \text {(10b)}\\ & \frac{dz}{dt}={-k}_1y_1+k_2y_2 \: \qquad \qquad \qquad \qquad \qquad \qquad \qquad \qquad \text {(10c)} \mathrm {\ } \end{array}\right. } \end{aligned}$$

In our model, we’ve introduced a new parameter *P* (ranging between *0 ≤ P < 1*) as discussed by Ramtani et al. (2023a, 2023b, 2023c). This addition allows us to explore different scenarios by adjusting how the tumor or pathophysiological modulation parameter impact bone remodeling. The parameter values and their potential biological implications are summarized in Table 1.

We explore nine scenarios (0–8) (see Table 1) that systematically perturb osteoclast/osteoblast production and removal rates $(\alpha _1,\beta _1,\alpha _2,\beta _2)$ through a pathophysiological modulation parameter variable *P∈ [0,1)*, where multiplying a rate by *(1-P)* denotes inhibition and dividing by *(1-P)* denotes stimulation. Scenario 0 is the unmodified physiological reference. Scenarios 1–4 implement paired, biologically plausible extremes: (1) resorption-biased (stimulated osteoclast rates; inhibited osteoblast rates), (2) formation-biased (inhibited osteoclast rates; stimulated osteoblast rates), (3) strongly lytic (*↑*OC production with *↓*OC removal, plus *↓*OB production with *↑*OB removal), and (4) the strongly formative inverse of (3). Scenarios 5–8 isolate single mechanisms–(5) *↑*OC production, (6) *↓*OC removal, (7) *↑*OB production, (8) *↓*OB removal–to separate recruitment from lifespan effects. Together, these cases cover clinically relevant tendencies (lytic vs. sclerotic) and enable sensitivity analyses of how specific rate changes alter cycle period and net mass balance.

Clinically, the scenarios map to recognizable phenotypes: **0** corresponds to normal adult remodeling (baseline). **1** models net bone loss states such as postmenopausal or glucocorticoid-induced osteoporosis and inflammatory osteolysis (e.g., rheumatoid arthritis, periodontitis). **2** mimics formation-dominant contexts such as the response to mechanical loading or anabolic therapy (intermittent PTH, anti-sclerostin), with net accrual. Scenario 2 represents a formation-dominant (net anabolic) regime consistent with intermittent PTH therapy. Although intermittent PTH transiently elevates both resorption and formation markers, the net outcome is bone gain because formation exceeds resorption (anabolic window). In this reduced BMU population model, Scenario 2 is therefore defined as a cycle-averaged net anabolic balance (enhanced osteoblast activity relative to osteoclast activity). **3** represents strongly lytic disease, typified by multiple myeloma and lytic bone metastases (breast, renal, thyroid). **4** reflects formative/sclerotic conditions–e.g., sclerotic metastases (prostate), late/sclerotic phase of Paget’s disease, or osteoclast-defect states such as osteopetrosis. **5** isolates increased osteoclastogenesis seen with high RANKL signaling (primary/secondary hyperparathyroidism, myeloma, immobilization). **6** isolates prolonged osteoclast survival, observed with estrogen deficiency or chronic glucocorticoid exposure. **7** isolates increased osteoblast recruitment as in early fracture healing, mechanical loading, or under anabolic agents. **8** isolates prolonged osteoblast survival/retention consistent with Wnt/*(β )*-catenin activation (sclerosteosis/van Buchem, LRP5 high-bone-mass) or anti-sclerostin therapy. These correspondences are heuristic; real diseases often combine multiple mechanisms.

In addition, Eq. (10c) tracks changes in bone mass, denoted as *z=z(t)*. Here, $k_i$ represents the normalized activity related to bone resorption and formation for *i=1* and *i=2*, respectively. The terms $y_i=y_i(t,P)$ describe the number of cells either breaking down or building bone.

This equation aligns with the model by Svetlana et al. (2003), measuring bone mass changes as a percentage of the initial mass based on the counts of osteoclasts and osteoblasts, as given by:

11 $$\begin{aligned} {2y}_i=\left( x_i-\overline{x_i}\right) + \mid \left( x_i-\overline{x_i}\right) \mid \end{aligned}$$

Additionally, ${\overline{x}}_i$ and $\overline{P}$ represent the steady-state numbers of cells and the tumor pathophysiological modulation parameter percentage, respectively (see for more details Ramtani et al. (2023c).

**Table 1:** Parameters for Different Scenarios

Scenario	$\widehat{\alpha _1(P)}$	$\widehat{\alpha _2(P)}$	$\widehat{\beta _1(P)}$	$\widehat{\beta _2(P)}$
0	$\alpha _1$	$\alpha _2$	$\beta _1$	$\beta _2$
1	$\frac{\alpha _1}{1-P}$	$\alpha _2(1-P)$	$\frac{\beta _1}{1-P}$	$\beta _2(1-P)$
2	$\alpha _1(1-P)$	$\frac{\alpha _2}{1-P}$	$\beta _1(1-P)$	$\frac{\beta _2}{1-P}$
3	$\frac{\alpha _1}{1-P}$	$\alpha _2(1-P)$	$\beta _1(1-P)$	$\frac{\beta _2}{1-P}$
4	$\alpha _1(1-P)$	$\frac{\alpha _2}{1-P}$	$\frac{\beta _1}{1-P}$	$\beta _2(1-P)$
5	$\frac{\alpha _1}{1-P}$	$\alpha _2$	$\beta _1$	$\beta _2$
6	$\alpha _1$	$\alpha _2$	$\beta _1(1-P)$	$\beta _2$
7	$\alpha _1$	$\frac{\alpha _2}{1-P}$	$\beta _1$	$\beta _2$
8	$\alpha _1$	$\alpha _2$	$\beta _1$	$\beta _2(1-P)$

#### Tumor with Komarova’s Structure

In our updated approach, and following our previous works Ramtani et al. (2023a, 2023c); Sánchez et al. (2024), we build on Komarova’s model by adding a new term that captures how tumors affect the rates of osteoclasts and osteoblasts. This enhancement modifies the original equations to include the tumor’s impact, introducing two new parameters, $g_{31}$ and $g_{32}$, to represent this influence. The revised equations now take the following form:

$$\begin{aligned} {\left\{ \begin{array}{ll} & \frac{dx_1}{dt}={{\upalpha }}_{\textrm{1}}x^{g_{11}}_1x^{g_{21}}_2P^{g_{31}}-{{\beta }_1x}_1 \qquad \qquad \qquad \qquad \qquad \qquad \qquad \text {(12a})\\ & \frac{dx_2}{dt}={\alpha }_2x^{g_{12}}_1x^{g_{22}}_2P^{g_{32}}-{\beta }_2x_2 \qquad \qquad \qquad \qquad \qquad \qquad \qquad \text {(12b}) \end{array}\right. } \end{aligned}$$

We explored one particular scenario, which can be adjusted by using various parameters. These parameters should be chosen outside the stability zone when dealing with cancer cases.

#### Tumor Affecting the Komarova’s Structure Through the Paracrine and Autocrine Parameters

Bruce P Ayati et al. (2010) developed a mathematical model inspired by Komarova’s approach. Their model adjusts paracrine and autocrine effects based on the density of tumor cells. They extended their model to work in two dimensions. Following (Ramtani et al. 2023a), we will explore two versions of a similar model: (1) the cancer cell model proposed by Bruce P Ayati et al. (2010), and (2) the new structure introduced in this paper. The specific equations used are detailed in (13).

$$\begin{aligned} {\left\{ \begin{array}{ll} & \frac{dx_1}{dt}={{\upalpha }}_{\textrm{1}}x^{\widetilde{g_{11}}}_1x^{\widetilde{g_{21}}}_2-{{\beta }_1x}_1 \qquad \qquad \qquad \qquad \qquad \qquad \text {(13a)}\\ & \frac{dx_2}{dt}={\alpha }_2x^{\widetilde{g_{12}}}_1x^{\widetilde{g_{22}}}_2-{\beta }_2x_2 \qquad \qquad \qquad \qquad \qquad \qquad \text {(13b)} \end{array}\right. } \end{aligned}$$

In the updated model, it was introduced (Ramtani et al. 2023a) four new parameters to better capture the impact of cancer on bone remodeling, compared to the original Komarova model. Specifically, we have:

$$\begin{aligned} \widetilde{g_{11}}(P)&= g_{11}\left( 1 + r_{11}\frac{P}{\sigma }\right) , \\ \widetilde{g_{21}}(P)&= g_{21}\left( 1 + r_{21}\frac{P}{\sigma }\right) , \\ \widetilde{g_{12}}(P)&= \frac{g_{12}}{1 + r_{12}\frac{P}{\sigma }}, \\ \widetilde{g_{22}}(P)&= g_{22} - r_{22}\frac{P}{\sigma }, \end{aligned}$$

where $r_{ij}$ are constants. These modifications allow the model to reflect how cancer affects the biochemical environment of bone remodeling. The term $r_{ij}\frac{P}{\sigma }$ introduces the coupling between tumor cells and osteoclasts/osteoblasts, adjusting the parameters $g_{ij}$ to better simulate cancer’s influence.

A detailed analysis of the model’s stability can be found in Bruce P Ayati et al. (2010), and in this paper, we apply a new methodology to revisit and validate these findings.

### Elastic Modulus Update

Using a similar approach to Hambli (2014), it is considered that the elastic modulus $E_0$ is modified by the percentage change in bone mass, denoted by *z*, obtained from Komarova’s equations and the accumulated damage equation, given by:

14 $$\begin{aligned} \frac{E}{E_0} = (1 - D_d)z \end{aligned}$$

It is important to highlight that this equation applies at the local level of the BMU. If the model is to be extended to a continuous domain, its value must be averaged accordingly. In the case of finite element analysis, a homogenization method should be employed to transition the model into the continuum.

### Methodology for Computing the Bone-BMU Accumulation Damage Model

The calculation of the damage accumulation model for bone-BMU can be carried out through the following steps:

0. Initialize values for osteoclasts ($x_1$), osteoblasts ($x_2$), bone mass (*z*), accumulated damage ($D_d$), and any pathological conditions (if present) related to bone via Komarova’s model (denoted as *P*) and through the fatigue model, i.e., $k_a$, $k_b$, or $k_c$.
1. In this step, Komarova’s model is computed, yielding the number of osteoclasts, osteoblasts, and bone mass. Additionally, any previously established damage models from prior works following (Ramtani et al. 2023b) can be included. The models used can follow one of the Eqs. (1), (10), (12), or (13).
2. Simultaneously, a stress state is generated randomly (or following a specific function) at a point over time, aligned with the temporal evolution of the BMU.
  2.1 If the load generation is based on sinusoidal functions, the values for mean stress $\sigma _m$ and alternating stress $\sigma _a$ will be known. At each time step, the value of $n_i = \frac{1}{2}$ is added to Miner’s equation (representing half a load cycle).
  2.2 If the load generation is based on random values, a Simple Moving Average (which gives $\sigma _m$) is calculated, and the random value is subtracted from the mean to obtain $\sigma _a$.
3. Using these values, the fatigue strength $S_f$ is computed via Gerber’s failure criterion, as described by Eq. (7).
4. The number of damage cycles $N_i$ required for $S_f$ to cause imminent damage is calculated using Eq. (8).
5. Miner’s rule is then applied, adding the term $\frac{n_i}{N_i}$, which for a time step is $\frac{1}{2N_i}$, using Eq. (9).
6. The sign of the osteoclasts’ slope ($x_1$) is calculated. If the value is negative, this means that osteoblasts will first form new tissue before destroying the existing tissue, i.e., the value of computed model/escenario is less than an threshold value ($\frac{dx_1}{t}<(x_1')_{opt}$), we can ensure that the BMU could remove the damaged tissue in order to re-establish and exchange (rebuilt) new tissue with zero damage. Thus, damage is removed, setting $D_d = 0$.
7. The elastic modulus is updated in conjunction with corrections for the damage and the quality of the present bone tissue. We use the Eq. (14).
8. The process returns to step 1 and begins again.

It is important to remark in Eq. 1, $\beta _1$ and $\beta _2$ are first-order removal rates (day$^{-1}$) of osteoclasts and osteoblasts, respectively; $\alpha _i$ are production terms, and $g_{ij}$ encode auto/paracrine regulation. Bone mass evolves as $dz/dt=-k_1y_1+k_2y_2$, where $y_i=\max \{x_i-\bar{x}_i,0\}$ are the positive deviations from reference cell levels. Mechanical loading enters through a fatigue-damage module: load histories are converted to mean/alternating stresses, corrected for mean stress, mapped to an S–N relation to obtain cycles to failure, and accumulated via Miner’s rule to yield mechanical damage $D_d$. This damage reduces local stiffness and, once a renewal criterion is met, triggers BMU (UBM) activity that removes the damaged packet (resetting $D_d$) and forms new bone. Hence, loads influence variation rates *indirectly*, by altering the damaged substrate and stiffness on which the BMU operates; the production/removal parameters $\alpha _i,\beta _i$ are not direct functions of load. We distinguish this mechanical pathway ($D_d$) from the biological “pathophysiological modulation parameter” *P* used in Table 1 to generate pathological scenarios by modulating $\alpha _i$, $\beta _i$, and/or $g_{ij}$.

In this work, the mechanical coupling is formulated as a *pointwise fatigue–damage law* acting at the BMU scale and driven by prescribed stress histories (sinusoidal, random, or step). Given the load history, we compute mean/alternating stresses, evaluate fatigue life via an S–N relation, and accumulate damage with Miner’s rule; the resulting mechanical damage $D_d$ updates the local stiffness according to $E/E_0=(1-P_d)\,z$, with *z* the bone-mass variable from Eq. (1). This stiffness update is defined at the *local* BMU level; to use it in a spatially resolved (continuum/FEM) setting, averaging and homogenization would be required. In our implementation, the ordinary differential equations are integrated in time per point/element (4th-order Runge–Kutta) without solving a global equilibrium PDE, so the mechanical part is D in the sense of being spatially uncoupled.

## Computational Model

The ordinary differential equations were addressed within each element using a 4th-order Runge-Kutta method. At every step of the computation, where the time increment is set to $\Delta t = 5 \:min$, the algorithm calculates the evolution of each cell within the extended BMUs. To ensure the accuracy of the simulation, previous numerical studies were conducted to determine the optimal value of *Δ t*, as explored in the comprehensive work by Ramtani et al. (2023) Ramtani et al. (2023c).

Notably, the initial conditions for osteoclasts, osteoblasts, and bone mass percentage were set to $x_1=11.586$ cells, $x_2=231.7238$ cells, and *z=95.5 %*, respectively, as reported in Ramtani et al. (b, c); Garzón-Alvarado and Linero (2012). In addition, we start with $D_d=0$

**Fig. 3:**
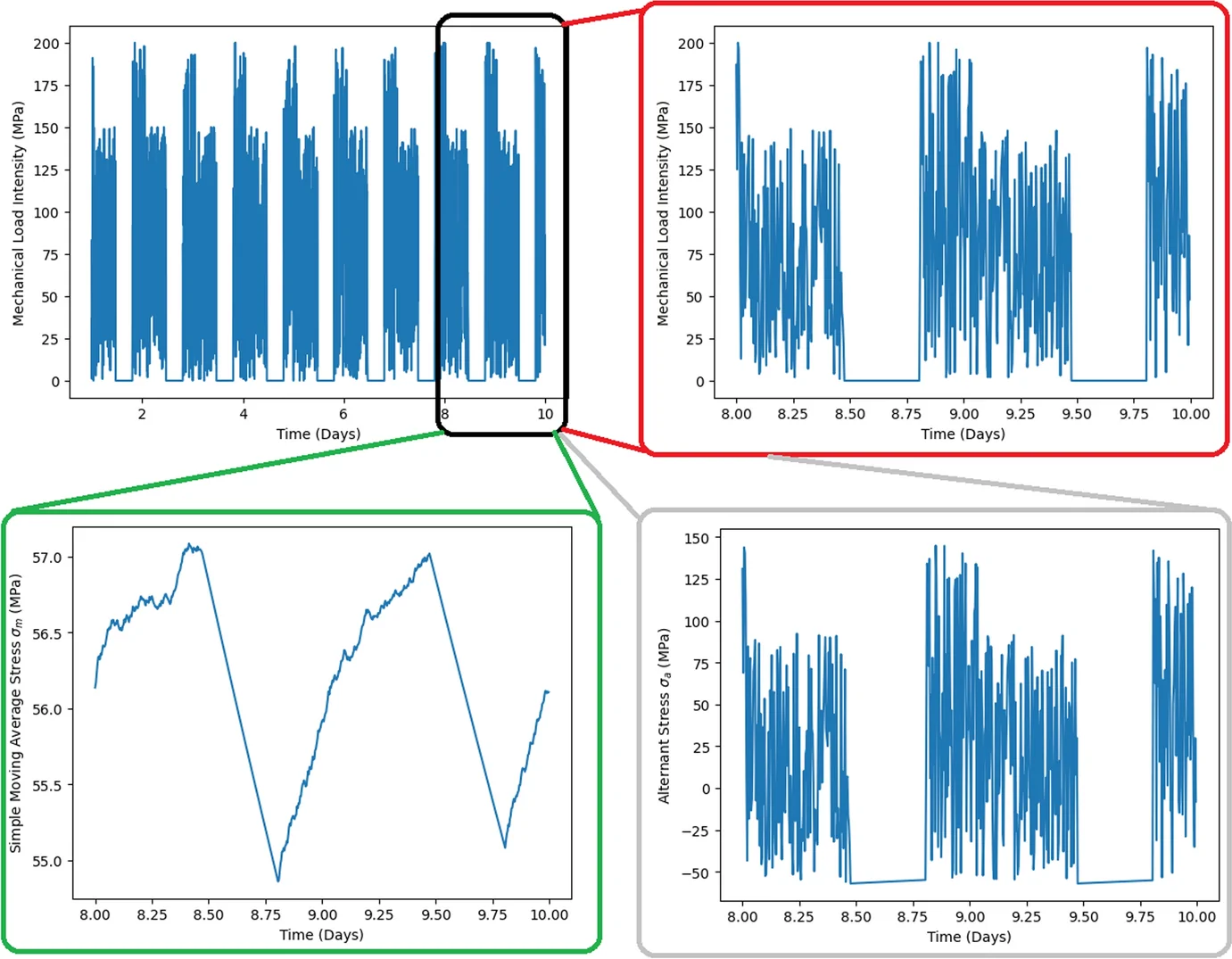
In the simulation, we have modeled 3000 days with random mechanical stress. The figure highlights only a 10-day period (top-left). On the top-right, a zoomed view of the last two days within this range is presented, showcasing key phases: a rest period, a peak during exercise, and regular daily activity. The bottom-left panel displays the average values over the entire simulation. Finally, the bottom-right panel illustrates the alternant stress, calculated based on the intensity of the mechanical load

Various levels of mechanical stress applied to different bones, such as the femur, vertebrae, tibia, and others, can be found in the literature. However, although these studies demonstrate a wide variability in the applied loads, we have chosen the value provided by Oftadeh et al. (2015); Hong Man Cho et al. (2022). We ran numerical simulations to see how the BMU reacts to different "imposed" loads that were chosen based on the stress levels reported in other works Oftadeh et al. (2015); Hong Man Cho et al. (2022). To impose the loads as realistically as possible, the day was divided into load periods, as shown in Figure 3 Whalen et al. (1988); Santalla et al. (2022).

This article proposes two types of signals: a random signal and a sinusoidal signal. For the random signal, three specific moments throughout the day were considered. The first moment corresponds to a resting or quiet state, where the tissue is unloaded. The second moment involves exercise, lasting two hours, during which the load increases. The third moment spans the remainder of the day, characterized by random activities, for which random loads are applied throughout this time. These three moments were modeled over the course of 3000 days, with each day containing these three phases. The random load varied between 0 and 200 megapascals.

The loading modes (assumed) were chosen considering the daily activity cycle of an average human who engages in daily exercise, structured as follows:

1. The cycle begins with a total rest activity phase (first subcycle), where no load is applied, $\sigma = \sigma _m = \sigma _a = 0$. This interval lasts for 96 steps of 5 minutes each, totaling 8 hours Santalla et al. (2022).
2. Following a workout routine, a random increased load cycle was imposed, ranging from *σ = 0* MPa to *σ = 200* MPa, considering an individual who exercises and experiences momentary overloads due to physical activities such as weightlifting or running. This subcycle lasts for 24 steps of 5 minutes, corresponding to 2 hours of exercise.
3. Finally, the last subcycle, consisting of various daily activities without overload, imposes a random load ranging from *σ = 0* MPa to *σ = 150* MPa. This subcycle takes into account activities such as walking, office work, and/or low-intensity physical labor Santalla et al. (2022).

**Fig. 4:**
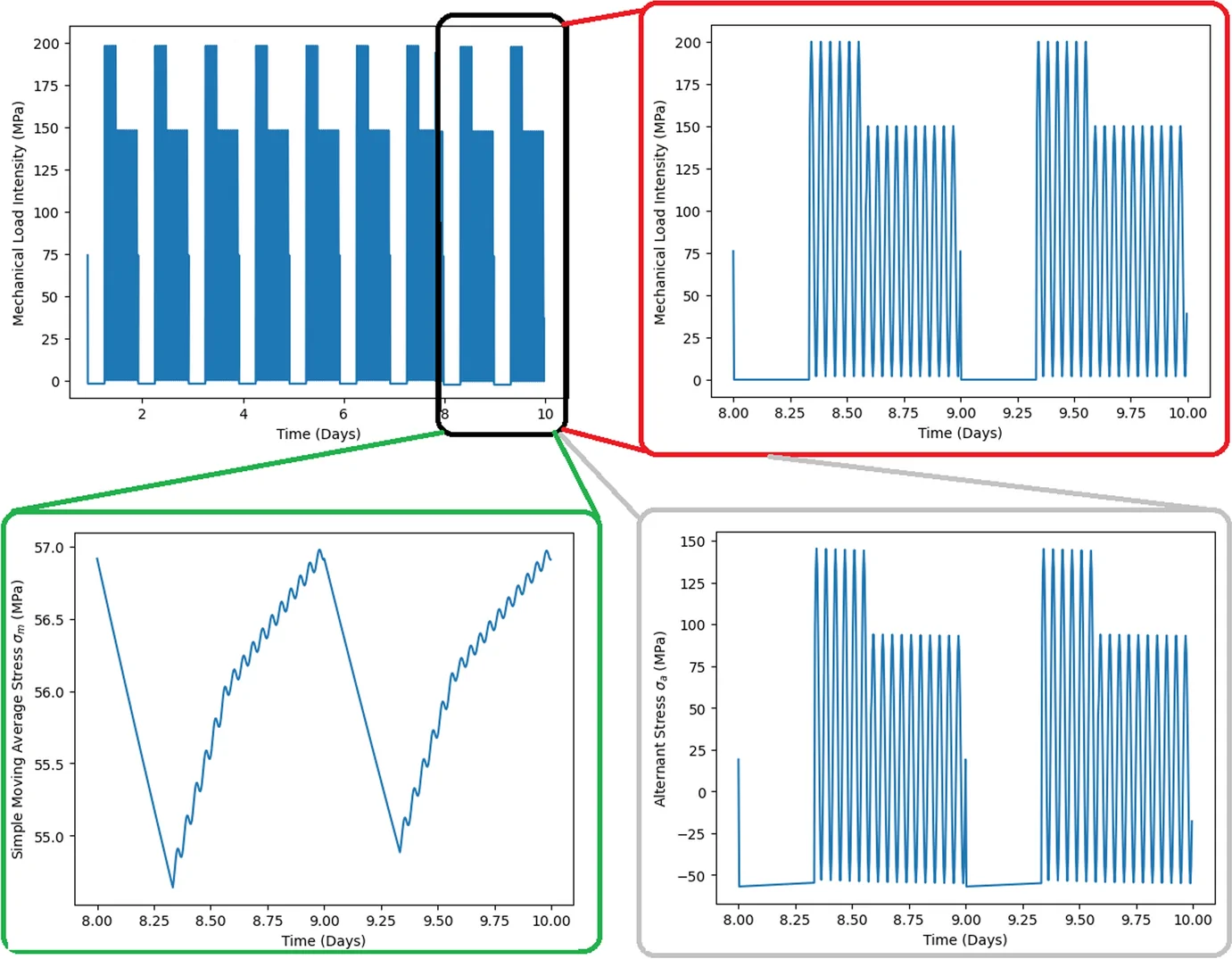
In the simulation, we have modeled 3000 days with sinusoidal mechanical stress. The figure highlights only a *10-*day period (top-left). On the top-right, a zoomed view of the last two days within this range is presented, showcasing key phases: a rest period, a peak during exercise, and regular daily activity. The bottom-left panel displays the average values over the entire simulation. Please note: Damage rises with daytime loading, is flat overnight, and drops only at renewal onsets (marked with vertical lines). Ten-day zoom (days) from the same simulation shown ahead. Daytime loading produces stepwise increases in cumulative damage $D_d$; overnight it remains flat. A drop in $D_d$ occurs only at renewal onset Finally, the bottom-right panel illustrates the alternant stress, calculated based on the intensity of the mechanical load.

The imposed load profiles can be observed in Fig. 3, which depicts the load cycle over 10 days (note that the total simulation spans 3000 days). Peaks of mechanical stress (during exercise hours), lower stress regions (low-intensity daily activity), and null stress (rest periods) are evident. On the right, a zoom-in is shown for an interval from day 10 to day 11, highlighting the random nature of the load and the differentiation between the previously mentioned subcycles. The zoomed-in section and the green box also show the calculation of the simple moving average of the mechanical load over a day, allowing us to obtain the mean stress, $\sigma _m$. In the lower right section, the alternating and oscillatory nature of the alternating load around the mean is visible, denoted as $\sigma _a = \sigma - \sigma _m$. Note that $\sigma _m$ is obtained by averaging the loads using the simple moving average method Anderson et al. (2020). It is important to remark that the calculation of the average and alternant load will be used in the Gerber equation. Additionally, it illustrates how the load signal was decomposed into mean load and alternating load. These two stress values–mean stress and alternating stress–will be used to understand the behavior of the load and the structural damage to the bone tissue, applying Gerber’s damage criterion. The figure also demonstrates how the alternating mechanical load and the mean load contribute to the development of structural damage over time.

To simulate the bone remodeling process, the possible scenarios described in Ramtani et al. (2023a, [Bibr CR29][Bibr CR30]) were selected. These scenarios took into account pathologies associated with the BMU’s behavioral changes according to Komarova’s model Svetlana et al. (2003). As a result, three possible models were considered as it was described in the section of "Modification of Komarova’s model". The parameters used in the simulation are listed in Table 2.

It is important to remark that following Komarova et al. Svetlana et al. (2003), we initialize the cell populations at the nontrivial steady state $(\bar{x}_1,\bar{x}_2)$ and trigger a remodeling event by a small, momentary increase in osteoclasts at $t{=}0$; only deviations above steady state ($y_i=\max \{x_i-\bar{x}_i,0\}$) contribute to resorption/formation. Bone mass is expressed relative to its initial value, so we set *z(0)=100%*. The accumulated mechanical damage is initialized as $D_d(0)=0$; it builds up from the prescribed load history and is reset during renewal, yielding a per-cycle terminal value $D_d=0$ %.

In addition, a sinusoidal load pattern was also applied, with variations in frequency (see Fig. 4). Similar to the previous case, the day was divided into an eight-hour rest period, a two-hour exercise period, and the remaining 14 hours with lower intensity movement compared to the exercise period. In this case, the frequency of the sinusoidal wave was varied, considering values of 60, 30, 20, and 10 Hz. Moreover, a random process was also taken into account in the analysis.

**Table 2:** Parameters used in this work

**Parameter**	**Action**	**Value**
${{\upalpha }}_{\textrm{1}}$	Osteoclast production rate	3 osteoblasts.day${}^{-1\ \ }$ Svetlana et al. (2003)
${{\upalpha }}_{\textrm{2}}$	Osteoblast production rate	4 osteoblasts.day${}^{-1}$ Svetlana et al. (2003)
${\beta }_1$	Osteoclast removal rate	0.2 osteoclasts.day${}^{-1}$ Svetlana et al. (2003)
${\beta }_2$	Osteoblast removal rate	0.02 osteoclasts.day${}^{-1\ \ }$ Svetlana et al. (2003)
$g_{11}$	osteoclast autocrine regulation	1.1 Svetlana et al. (2003)
$g_{21}$	osteoblast-derived paracrine regulation	−0.5 Svetlana et al. (2003)
$g_{12}$	osteoclast-derived paracrine regulation	1 Svetlana et al. (2003)
$g_{22}$	osteoblast autocrine regulation	0 Svetlana et al. (2003)
$k_0$	Normalized activities of bone formation/resorption	1.0 % osteoclasts${}^{-1}$day${}^{-1}$
$k_1$	Normalized activities of bone resorption	0.093 % osteoclasts${}^{-1}$day${}^{-1}$ Svetlana et al. (2003)
$k_2$	Normalized activities of bone formation	0.0008 % osteoblasts${}^{-1}$day${}^{-1}$ Svetlana et al. (2003)
*P*	Pathophysiological Modulation Parameter	0.05 dimensionless[used in all the models here presented, except second model tested, with 0.10,0.20,0.40] (computed)
**Parameters for second model ** Ramtani et al. (2023c)		
$g_{d1}$	Disease-derived paracrine regulation	−0.1 Ramtani et al. (2023b,c, )
$g_{d2}$	Disease-derived paracrine regulation	0 Ramtani et al. (2023b, c)
**Parameters for second model** Bruce P Ayati et al. (2010)		
$r_{11}$	Parameter afecting osteoclast-autocrine factor	0.05 Bruce P Ayati et al. (2010)
$r_{21}$	Parameter afecting osteoblast-paracrine factor	0.0 Bruce P Ayati et al. (2010)
$r_{12}$	Parameter afecting osteoclast-paracrine factor	0.0 Bruce P Ayati et al. (2010)
$r_{22}$	Parameter afecting osteoblast-autocrine factor	0.2 Bruce P Ayati et al. (2010)

## Results and Discussion

### Model 0: Damage Accumulation in the Presence of Normal Physiological BMU Behavior

**Fig. 5:**
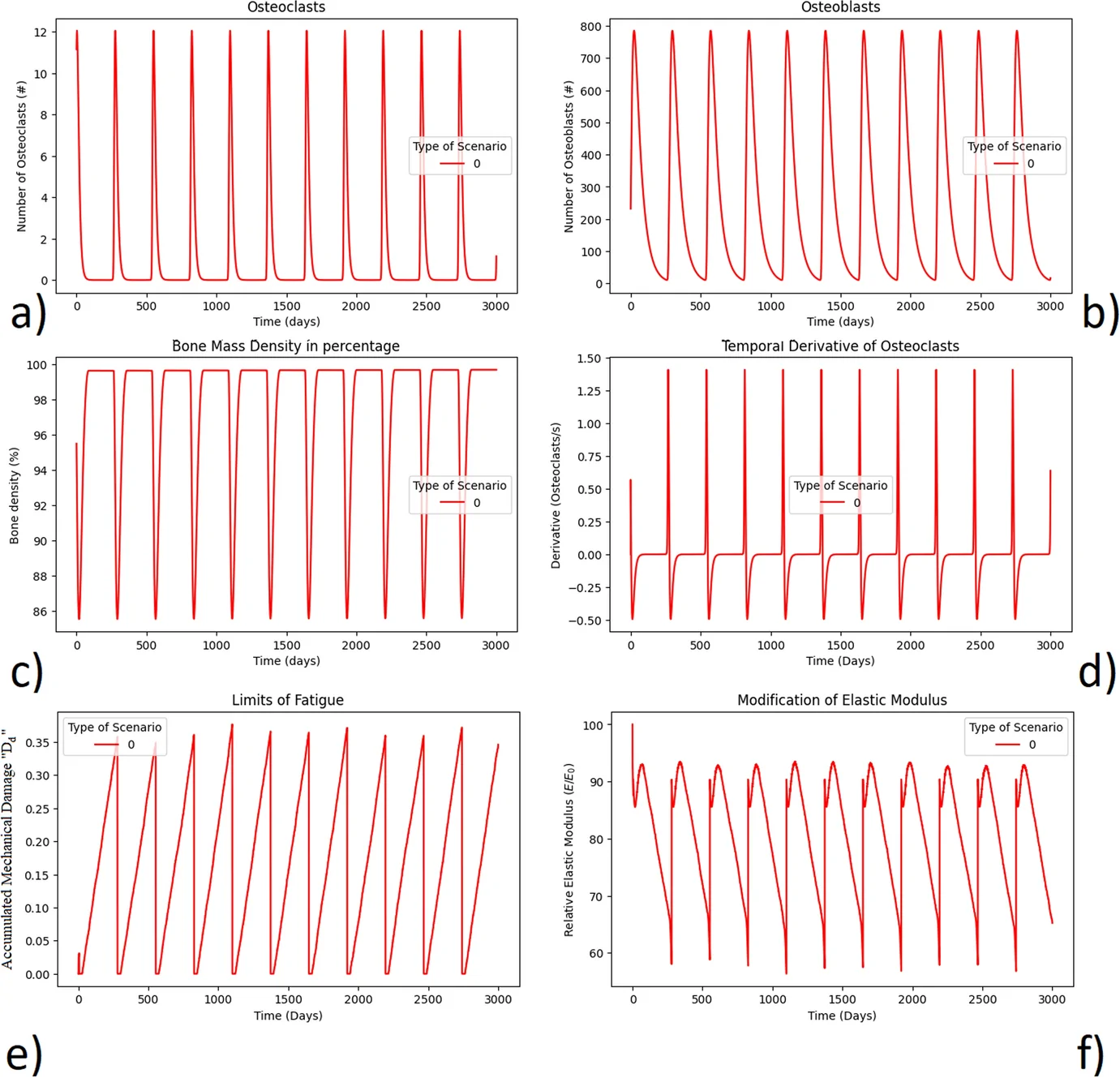
Physiological BMU dynamics under random daily stress (Model 0). (**a**) Osteoclast level $x_1(t)$; (**b**) osteoblast level $x_2(t)$; (**c**) bone-mass fraction *z*(*t*); (**d**) osteoclast-mediated mass turnover; (**e**) accumulated mechanical damage $D_d(t)$, which peaks under stochastic loading and is reset to zero at renewal; (**f**) normalized stiffness $E/E_0=(1-D_d)\,z$, showing a transient decline before each renewal and recovery thereafter. Eleven remodeling cycles are observed over *∼*3000 days.

The BMU (Bone Multicellular Unit) is simulated without any damage or parameter changes so that the behavior of the model could be understood in its natural physiological state. This allows us to observe the model’s response to the imposed loads mentioned previously. In Fig. 5, it shows the evolution of osteoclasts, osteoblasts, and bone mass. This evolution is physiological, maintaining the cycle without any alteration in both its operating period and the amplitude of the wave. Specifically, osteoclasts reach values of up to approximately 12 cells, and osteoblasts up to approximately 800 cells, before returning to null values where the bone remodeling process restarts.

In Fig. 5, the first row illustrates the variation of osteoclast activity over time. This variation, a key characteristic of bone remodeling, allows for the continuous renewal of bone tissue, removing microfractures and other types of minor structural damage. This process helps maintain the structural integrity of the tissue, enabling it to respond to mechanical loading. The remodeling period spans, approximately, 250 days, during which 11 cycles of bone mass renewal occur.

In the second row, the variation of bone mass over time is shown, revealing a plateau followed by renewal phases where bone mass decreases and is subsequently replaced. To the right of this second row, the temporal variation of osteoclast-mediated mass turnover is depicted, highlighting the role of osteoclasts in leading the process of tissue breakdown.

It is important to note that the damage accumulation graph appears as a ramp with undulations, reflecting the random nature of the load signal (Fig. 5). This damage accumulation dissipates completely when osteoclasts begin the bone renewal process, which is mediated by the osteoclast differential equation. This equation causes a sharp drop in the slope of the osteoclast count at the moment of renewal.

Finally, the third row presents the accumulation of damage over time, showing how damage builds up with random peaks (approximately, $D_d=0.35$) due to the stochastic nature of the applied loads. However, this damage decreases during the renewal process, resulting in a final accumulated damage, in each cycle, value of 0. The graph on the far right shows the variation in bone stiffness over time, illustrating how mechanical properties deteriorate drastically at a certain point before the renewal phase begins.

### Tumor Affecting the Activity of Cell Production or Removal:

#### Scenarios 1, 4, 7, and 8: Damage Accumulation in the Presence of Factors that Alter Remodeling Cycle Period

**Fig. 6:**
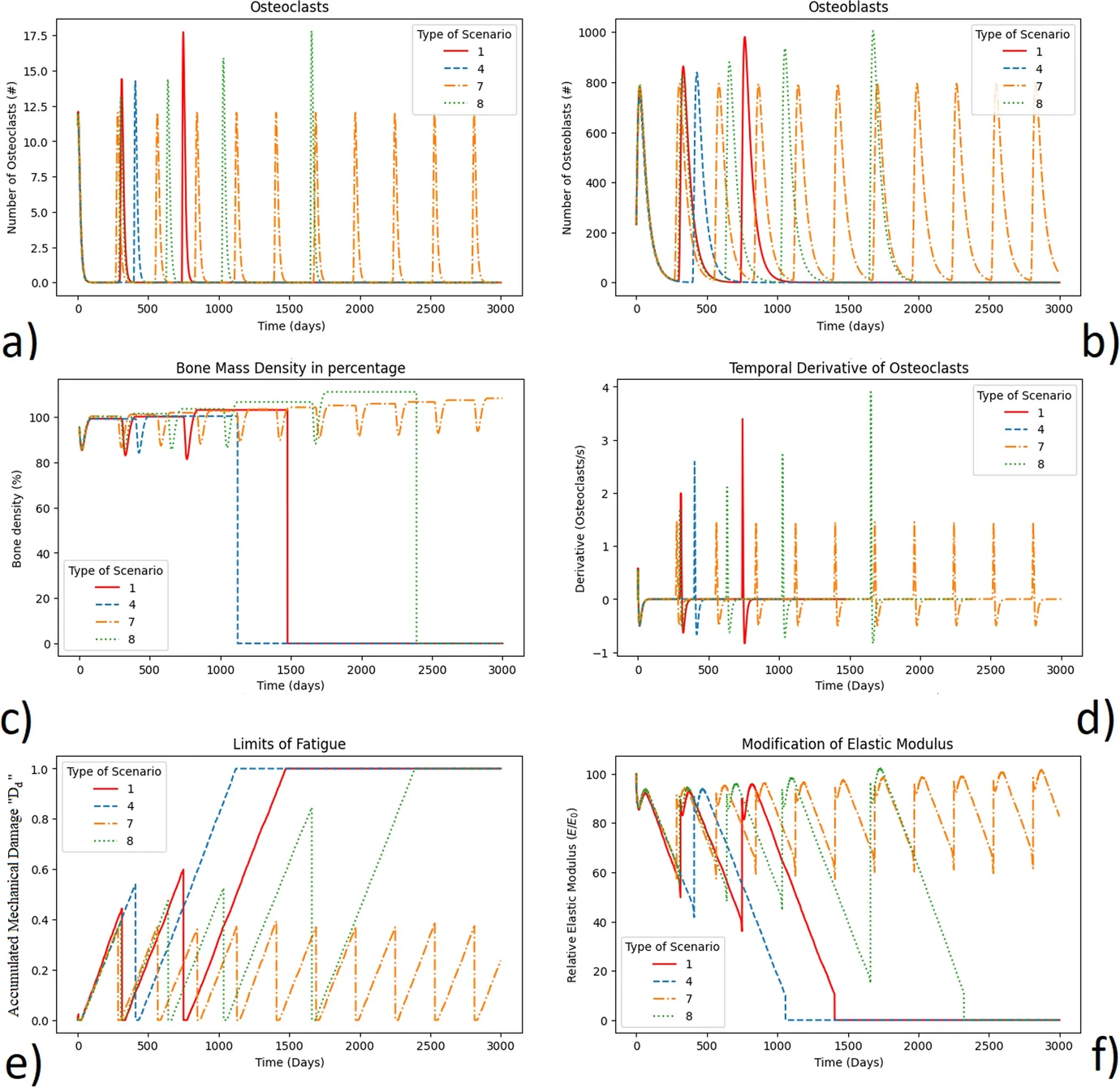
The figure illustrates the physiological evolution of osteoclasts, osteoblasts, and bone mass in the BMU model under pathological damage (scenarios 1, 4, 7, and 8). The first row shows osteoclast activity over 250 days, highlighting 11 bone remodeling cycles. The second row presents bone mass variation, with renewal phases and osteoclast-mediated mass turnover (right). The third row displays damage accumulation with random peaks due to stochastic loads, which dissipate during bone renewal, reducing final damage to zero. On the right, bone stiffness variation shows a decline before the renewal phase. (*P=0.025*).

In Fig. 6, various scenarios are presented based on the original Komarova’s model (Svetlana et al. 2003). These scenarios incorporate modifications on the the original Komarova’s equation (see Table 1), including biological modulation parameter caused by factors such as cancer, tumors, aging, and alterations in the bone remodeling cycle, such as osteoporosis. This biological alteration is represented by a parameter called " pathophysiological modulation parameter (*P=0.025*)." Depending on the type and level of biological alteration, following (Ramtani et al. 2023b, c), it affects the remodeling cycle differently. Particularly, these scenarios are centered in the bone remodeling cycle period alteration, especifically, increasing period time.

In scenario one, shown in red, the bone remodeling cycle period increases (from *T=250  days* to *T=500  days*, approximately) its duration. This extended period can be attributed to conditions like osteoporosis, where the remodeling cycle becomes much longer, allowing damage to accumulate in the tissue. During the first cycle, the damage is successfully removed. However, in the second cycle, damage increases slightly, and by the third cycle, the extended duration leads to insufficient removal of accumulated damage, resulting in structural failure. Structural failure occurs when the mechanical damage value reaches one, which signifies total failure of that particular tissue segment. This leads to the loss of the remodeling cycle, along with a depletion of osteoclast and osteoblast cells, as well as a significant reduction in material properties, potentially to the point where the tissue can no longer bear any load.

In Scenario 4, osteoclast activity is reduced (inhibited osteoclasts), while osteoblast activity is enhanced, resulting in a strongly formative remodeling response. As the damage accumulates gradually in the tissue, the blue line in the pathological damage level graphs shows how this leads to rapid structural damage over time (in *t=1000  days*), eventually culminating in complete structural failure of the bone tissue.

Scenario eight follows a similar pattern, where the remodeling cycle period increases, leading to structural damage. In this particular model, scenario eight also demonstrates a slight increase in bone mass due to an imbalance between osteoblasts and osteoclasts, where osteoblast activity exceeds that of osteoclasts. As a result, there is an increase in bone mass and a corresponding increase in the elastic modulus of the tissue. On the other hand, scenario seven resembles a more physiological condition, where tissue and mechanical damage are continuously removed during the remodeling cycle. This allows for the regular removal of accumulated damage, ensuring the survival and health of the bone tissue.

#### Tumor Affecting the Activity of Cell Production or Removal: Scenarios 2, 3, 5, and 6: Changes in Osteoblast and Osteoclast Behavior Affecting Bone Mass Production

**Fig. 7:**
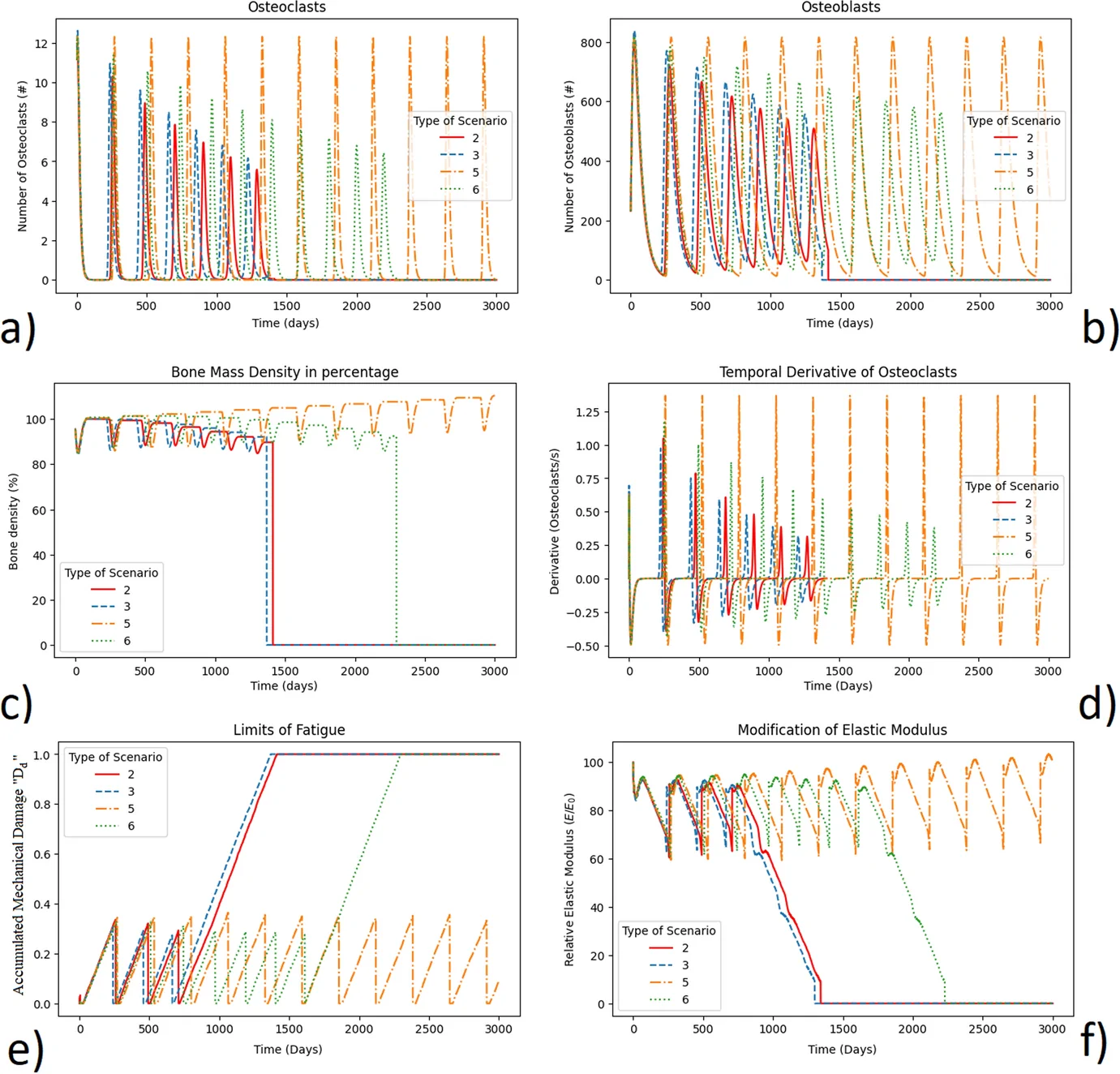
This figure compares physiological and pathological bone remodeling scenarios, highlighting the dynamics of osteoclasts and osteoblasts (first row) affecting bone mass (second row). Figure depicts scenarios 2, 3, and 6, where decreased osteoclast and osteoblast activity leads to defective turnover, simulating myeloma and osteoporosis. In contrast, scenarios 5 and 6 show increased osteoblast activity resembling osteosarcoma, where bone mass increases without adequate turnover. Scenario 5 demonstrates improved outcomes with higher osteoblast levels, likely from healthy exercise, resulting in less accumulated damage compared to the original physiological model. *P=0.025*.

In these scenarios, changes in the behavior of osteoblasts and osteoclasts are observed mainly, which in turn affect bone mass production. In Scenarios 2, 3, and 6, the number of osteoclasts and osteoblasts decreases over time, although some cells remain active in the tissue. This results in a defective process of bone turnover, where effective renewal of bone mass does not occur (see Fig. 7). All the simulations are performed using *P=0.025*.

In Scenario 2 and 3, there is a marked decrease in bone mass due to a relative increase in osteoclasts compared to osteoblasts, which may mimic the processes of myeloma and osteoporosis. In Scenarios 5 and 6, on the other hand, bone mass goes up because there are more osteoblasts than osteoclasts. This could be like osteosarcoma (Lim et al. 2022), where bone mass goes up a little but bone tissue does not turn over or renew itself enough (see Fig. 7).

In these cases, the slope of the osteoclast function does not reach the required value for effective turnover, leading to the accumulation of damage until failure occurs. In Scenarios 2, 3, and 6, damage begins to rise around days 750, 700, and 1600, respectively, ultimately reaching imminent failure at approximately days 1200, 1250, and 2200 days, approximately. The elastic modulus also decreases, leading to a total loss of the bone’s load-bearing capacity.

On the other hand, Scenario 5 (Fig. 7) shows a relatively higher number of osteoblasts, which may result from conditions such as regular and healthy exercise. This situation provides some protection against mechanical loads, slightly reducing the accumulated damage compared to the original physiological model by Komarova (see Fig. 7).

### Tumor with Komarova’s Structure: Imbalance in Osteoclasts and Osteoblasts

**Fig. 8:**
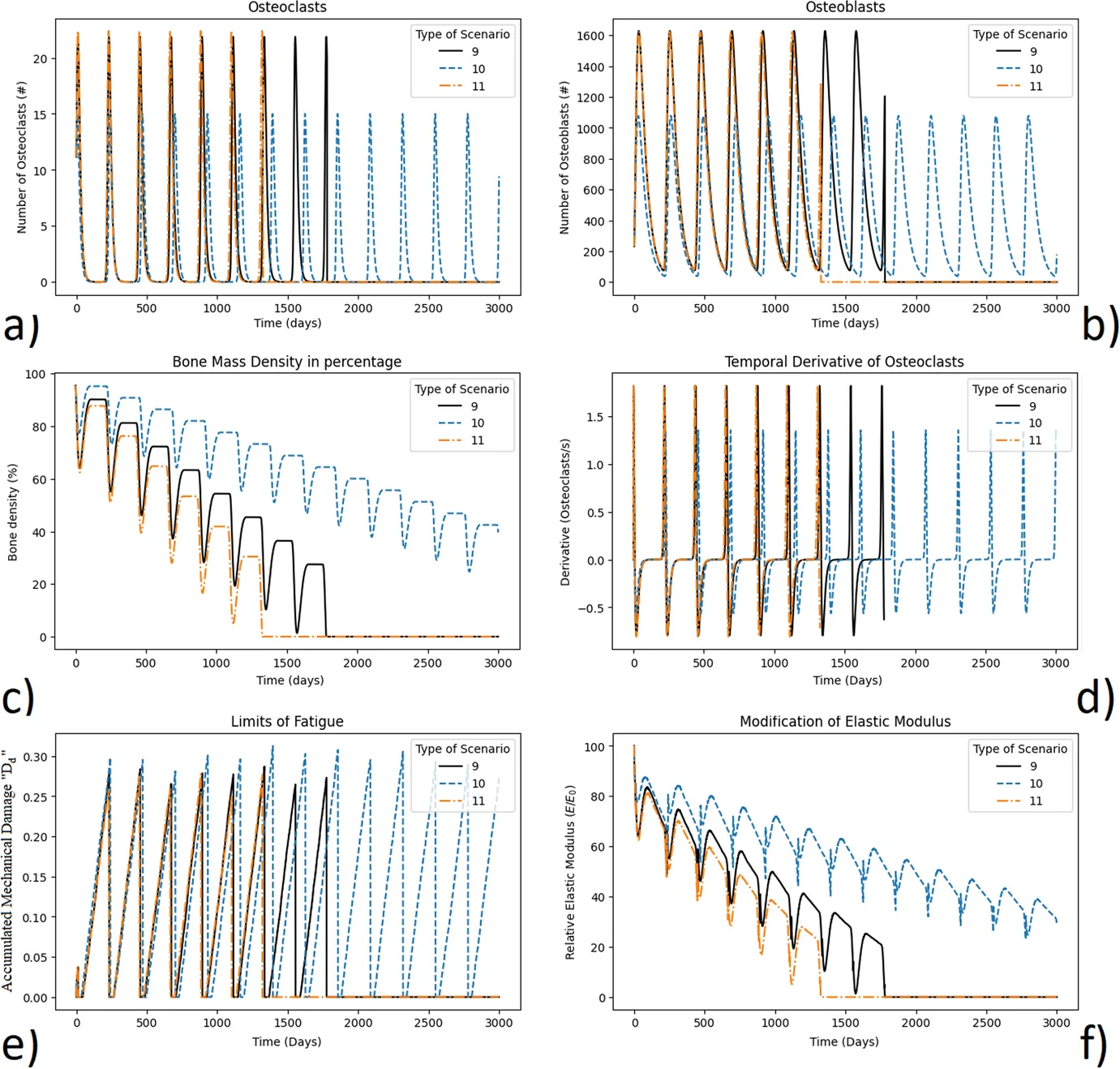
Same distribution than previous figures. Here, the figure presents results for a model with *P=0.025* across three scenarios: 9 ($g_{31}=-0.10$, $g_{32}=0.00$), 10 ($g_{31}=-0.05$, $g_{32}=0.00$), and 11 ($g_{31}=-0.10$, $g_{32}=-0.01$). Scenarios 9 and 11 show a greater imbalance in osteoblast and osteoclast quantities, where osteoclasts consume more bone tissue without effective replacement, leading to structural damage. Scenario 10 also demonstrates bone mass reduction, likely due to tumor conditions or osteoporosis; however, turnover remains effective, and the tissue continues to function, albeit with declining mechanical properties. This decline results from both external loading conditions and the biological processes of bone tissue generation.

Figure 8 shows the results for a model which uses *P=0.025* and for the scenario 9: $g_{31}=-0.10$, $g_{32}=0.00$; scenario 10: $g_{31}=-0.05$, $g_{32}=0.00$; and for scenario 11: $g_{31}=-0.10$, $g_{32}=-0.01$ in Equations (12). In cases nine and eleven, it is observed that the quantity of osteoblasts and osteoclasts are imbalanced in comparison with case ten. In those cases, osteoclasts are consuming a greater amount of bone tissue but are not effectively replacing the bone mass. This results in structural damage due to the imposed loading conditions, where the bone tissue first breaks down and the remodeling process ceases to function. This situation may be caused, for example, by osteoporosis or some type of tumor, which disrupts normal turnover, consistently reducing the amount of bone tissue present.

In scenario number 10, a process of bone mass reduction is also demonstrated, possibly due to tumor conditions or osteoporosis. Although the turnover is effective, bone mass decreases over time. Despite the accumulation of damage leading to the near disappearance of bone tissue in cases nine and eleven; in case ten, the tissue does not disappear and continues to function, albeit with reduced mechanical properties. This results in a gradual decline in mechanical properties, not only due to external mechanical conditions imposed by the previously described random loads but also because of the biological process of bone tissue generation itself.

### Tumor affecting the Komarova’s Structure Through the Paracrine and Autocrine Parameters

**Fig. 9:**
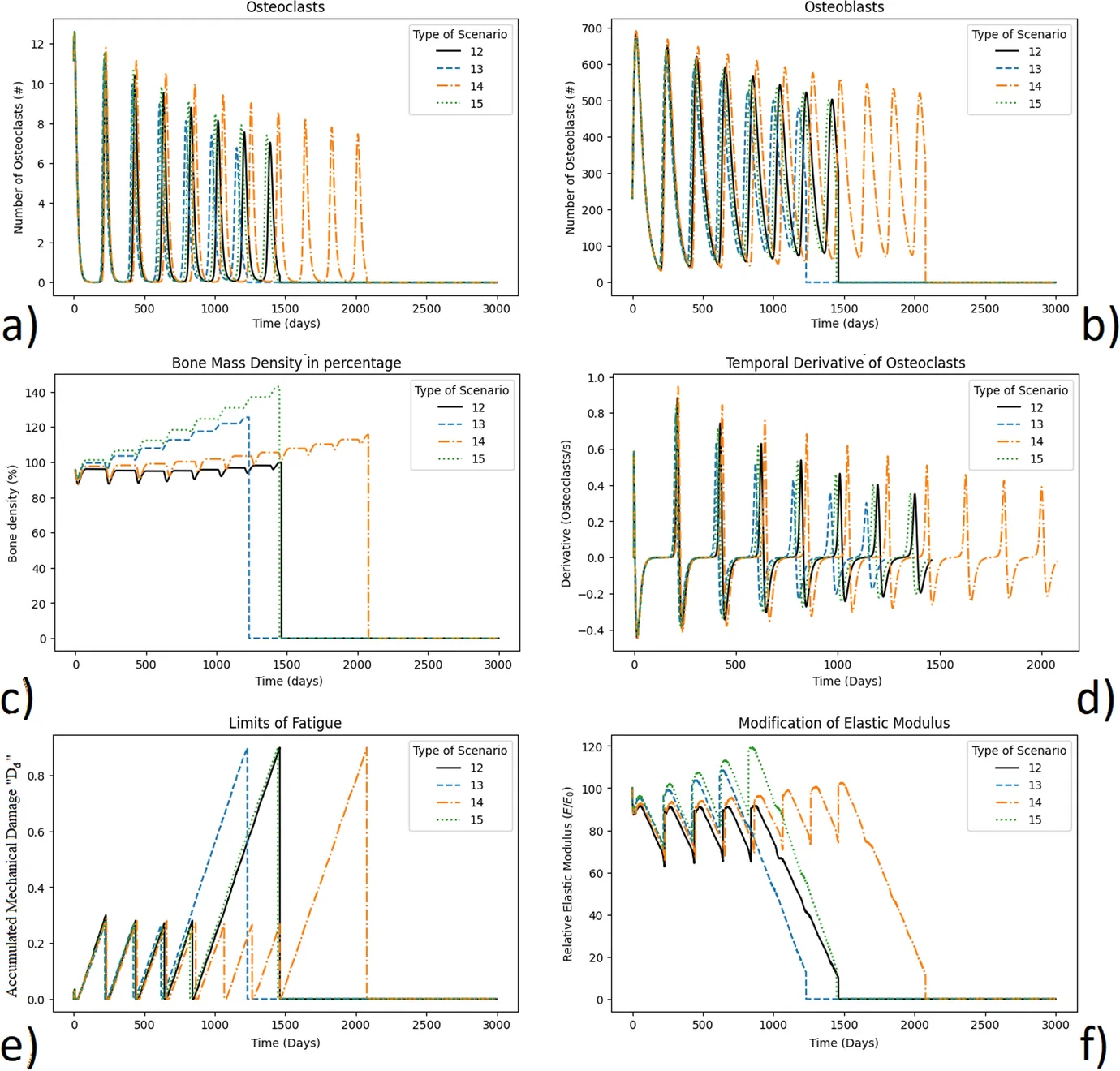
Same distribution of information than previous figures. This figure compares physiological and pathological bone remodeling scenarios, highlighting the dynamics of osteoclasts and osteoblasts affecting bone mass. The first row shows the natural evolution of these cells. Additionally, scenarios 12 ($r_{11} = 0.005$, $r_{22} = 0.2$), 13 ($r_{11} = 0.005$, $r_{22} = 0.25$), 14 ($r_{11} = 0.010$, $r_{22} = 0.2$), and 15 ($r_{11} = 0.010$, $r_{22} = 0.25$) illustrate variations in bone remodeling parameters, further emphasizing the influence of osteoblast and osteoclast dynamics on bone mass production.

Similarly, in scenarios 12, 13, 14, and 15, conditions arise where there is an imbalance between osteoblasts, leading to a slight increase in bone mass (see Fig. 9). However, the osteoclasts do not function effectively, resulting in a process of mechanical damage accumulation that can lead to structural collapse.

In these cases, the bone remodeling process fails, as seen in the second row of the figure see Fig. 9 (right side). This occurs because the turnover and osteoclasts lack sufficient efficacy to remove damaged tissue, allowing damage to persist despite the presence of increased bone mass (though with remaining damage in the structure). This results in damage $D_d = 1.0$ reaching its maximum value. This phenomenon can occur in multiple myeloma, as reported by Bruce P Ayati et al. (2010)

Below are the corresponding values for each scenario:

- **Scenario 12:** $$r_{11} = 0.005$$, $$r_{22} = 0.2$$
- **Scenario 13:** $$r_{11} = 0.005$$, $$r_{22} = 0.25$$
- **Scenario 14:** $$r_{11} = 0.010$$, $$r_{22} = 0.2$$
- **Scenario 15:** $$r_{11} = 0.010$$, $$r_{22} = 0.25$$

### With Sine Function as a Load Wave Form

#### Frequency Response

**Fig. 10:**
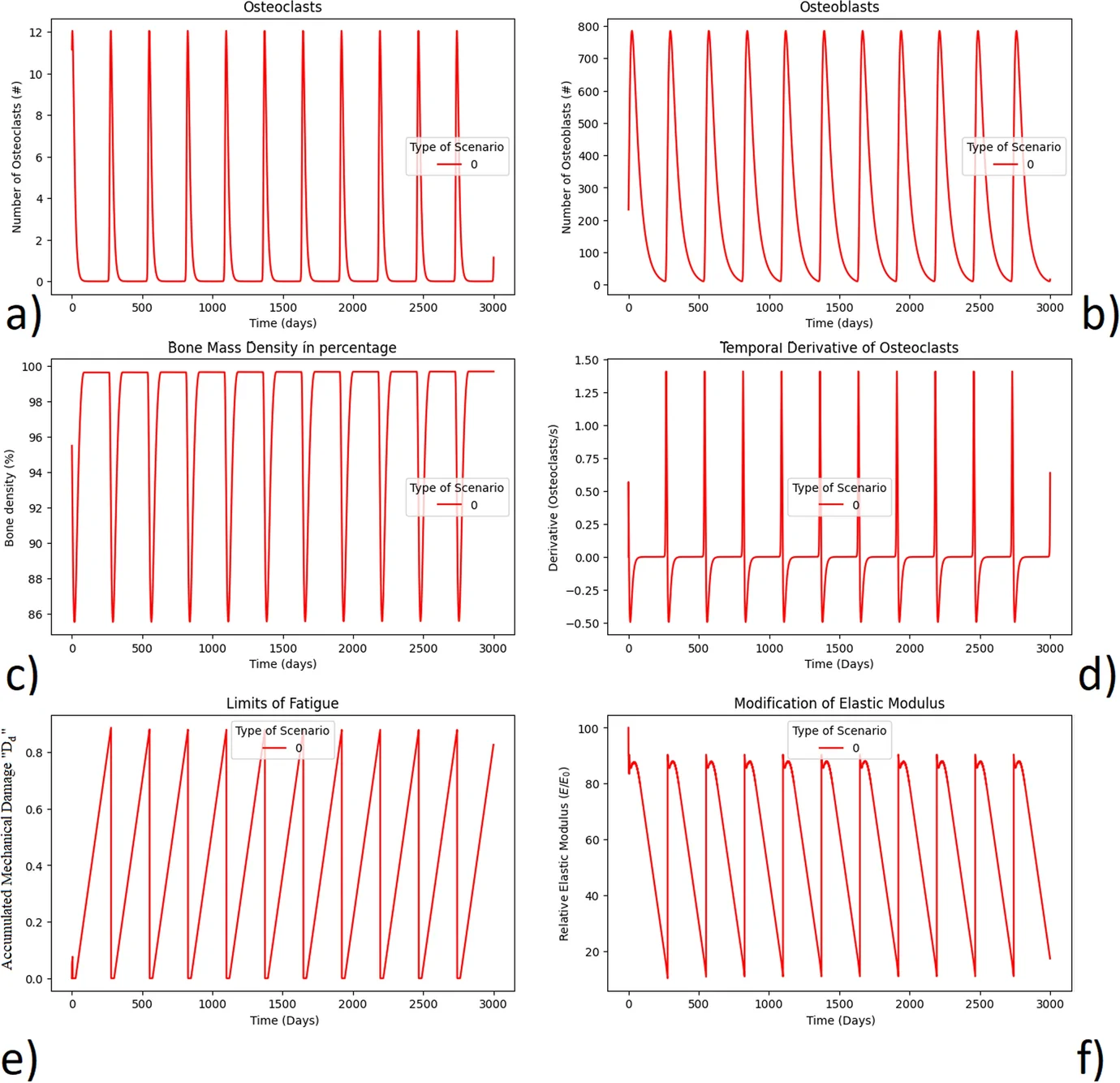
(**a**) Osteoclast and (**b**) osteoblasts activity over time; (**c**) bone mass variation; (**d**) osteoclast-mediated mass turnover; (**e**) damage accumulation over time, and (**f**) bone stiffness variation, at 48 Hz

In Fig. 10, we included cases with sinusoidal loads instead of random loads, as shown in previous examples. Specifically, in this graph, we show a sinusoidal load of 48 Hz. It can be observed that its response is similar to the random process, indicating that tissue turnover and renewal occur after a certain number of cycles in each remodeling phase. This is consistent with the physiological behavior reported for random loads.

It is important to note that the process can be described either sinusoidally or randomly, as discussed earlier (Computational Model Section).

### Modification of $S_{ut}$

**Fig. 11:**
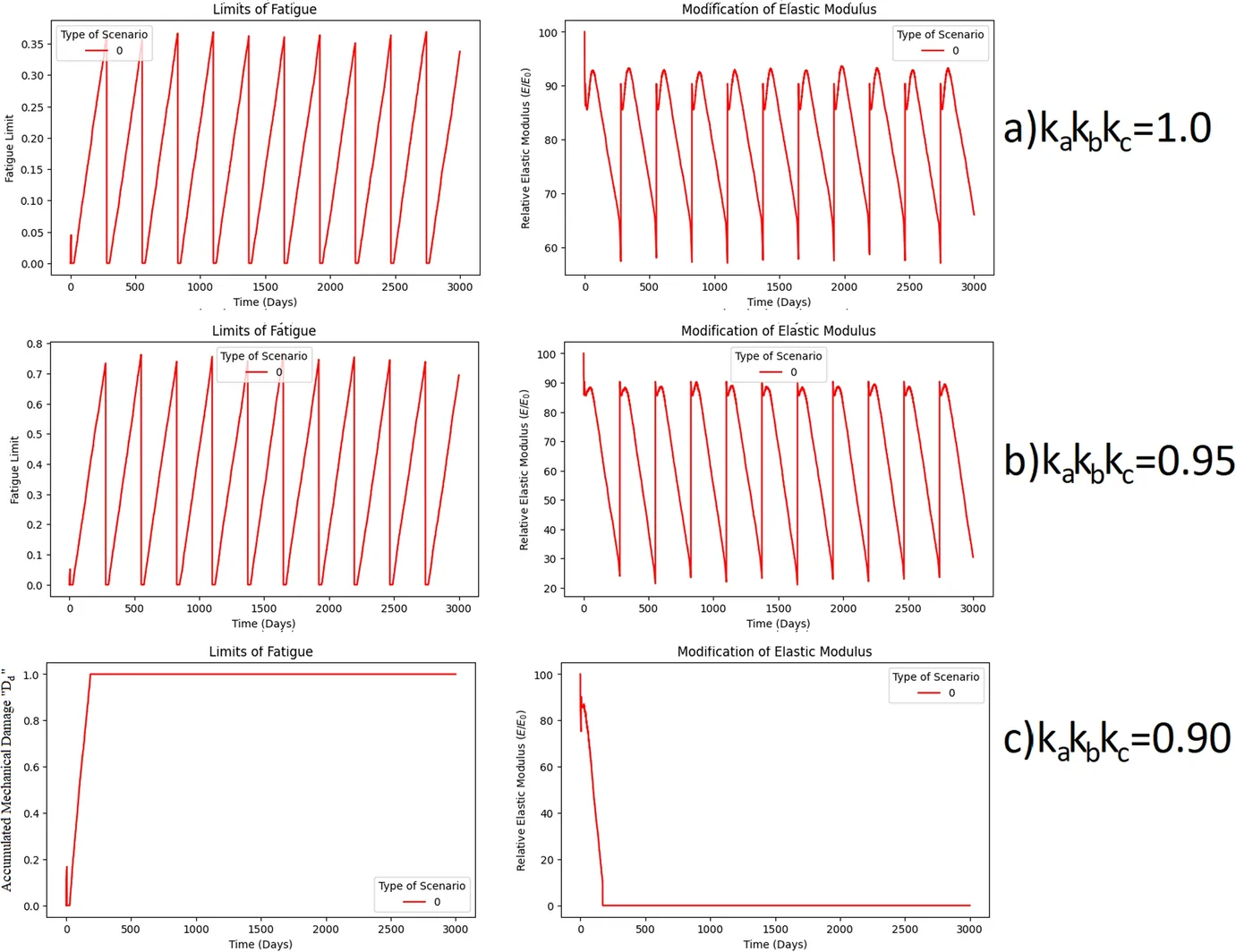
Row (**a**) No reduction of strenth, (**b**) reduction of 5% and (**c**) reduction of 10%. On the left: accumulated damage; On right: elastic modulus in time. The parameters are the same then the physiological case

Finally, as a proposal, values such as $k_a$, $k_b$, and $k_c$ could be used to reduce the ultimate strength, following a similar approach to that used in engineering design Shigley and Budynas (2019). For this reason, it is evident that lowering the ultimate stress value will inevitably lead to structural damage, as shown in the Fig. 11. In this figure, it is clearly observed that the reduction of these values accelerates the damage process. However, these parameters must be thoroughly studied in future research, especially if this type of algorithm is to be used to establish the maximum mechanical load relationships that a person’s bone tissue can withstand, considering their metabolic, physiological, and age-related characteristics.

## Conclusion

This work presents a preliminary computational framework that couples a classical BMU-scale remodeling model (Komarova-type osteoclast–osteoblast population dynamics) with a fatigue-based mechanical damage module. The primary contribution is methodological: we demonstrate how an S–N/Miner-type accumulation law can be embedded into a time-resolved remodeling setting so that mechanical degradation and biologically driven renewal can be explored within a single, internally consistent dynamical scheme. The results are hypothesis-generating and should be interpreted as qualitative BMU-local trends rather than as clinically actionable predictions.

Under physiological conditions, the coupled model reproduces stable remodeling cycles in which osteoclast-mediated resorption and osteoblast-mediated formation remain balanced, preventing uncontrolled degradation of bone mass fraction and stiffness over time. When cyclic loading histories are imposed (in this study restricted to moderate activity conditions within our loading representation), the framework captures the expected trend that repeated loading can progressively increase accumulated mechanical damage $D_d(t)$, and that the effective modulus decreases accordingly through the local update relation that combines damage and bone mass fraction. These outputs support the conceptual picture that fatigue-induced microdamage, if not sufficiently renewed, degrades mechanical competence, while targeted renewal can partially restore it.

Across the set of perturbed remodeling scenarios (implemented through controlled parameter modifications in the BMU kinetics), the simulations illustrate how shifts in osteoclast/osteoblast regulation can move the system away from homeostasis and alter cycle characteristics, bone mass fraction, and stiffness trajectories. Importantly, these scenario explorations are intended to demonstrate the responsiveness of the coupled framework and to provide mechanobiological interpretations at the BMU scale; they do not constitute validated disease modeling or quantitative prognosis.

Several limitations must be emphasized. First, the model is 0D (BMU-local) and does not solve spatial mechanics, load redistribution, or heterogeneous tissue adaptation; stresses are prescribed rather than emerging from a coupled continuum equilibrium problem. Consequently, the reported modulus and damage trajectories are local outputs and cannot be directly generalized to organ-scale behavior without spatial modeling and homogenization. Second, the fatigue component is implemented in a simplified form and, in the present study, is restricted to compressive loading and does not incorporate tension–compression asymmetry, anisotropy, or direction-dependent damage parameters. Third, stiffness is linked to bone mass fraction (and, where applicable, mechanical damage) but does not include mineralization kinetics, which can significantly affect elastic properties over distinct timescales. Finally, the “pathological” parameter settings represent phenomenological perturbations rather than data-driven estimates, and the framework is not yet calibrated or validated against experimental fatigue datasets or histomorphometric remodeling metrics. For these reasons, no clinical claims are made: the framework is not validated for patient-specific inference, fracture-risk prediction, or therapeutic decision support at this stage.

Future work will focus on: (i) adding a no-exercise (baseline/no-activity) control condition and performing systematic comparisons against the moderate-activity cases reported here, to isolate the specific contribution of exercise to $D_d(t)$ and *E*(*t*); (ii) extending the model to spatially resolved 2D/3D continua (e.g., FEM-based implementations) to capture load redistribution, heterogeneous renewal, and multi-BMU interactions; (iii) incorporating tension–compression asymmetry, anisotropy, and improved damage laws as data allow; (iv) introducing mineralization/demineralization dynamics so that modulus reflects both mass and mineral content; and (v) carrying out rigorous calibration, validation, and benchmarking against published mechanobiological models and experimental datasets before drawing quantitative or clinically oriented conclusions.

## Data Availability

No datasets were generated or analysed during the current study.

## References

[CR1] Anderson DR, Sweeney DJ, Williams TA, Camm JD, Cochran JJ (2020) Statistics for business and economics, 14th edn. Cengage Learning, Boston, MA

[CR2] Ayati BP, Edwards CM, Webb GF, Wikswo JP (2010) A mathematical model of bone remodeling dynamics for normal bone cell populations and myeloma bone disease. Biol Direct 5(1):1–17 20406449 10.1186/1745-6150-5-28 PMC2867965

[CR3] Bonfoh N, Novinyo E, Lipinski P (2011) Modeling of bone adaptative behavior based on cells activities. Biomech Model Mechanobiol 10(5):789–798. 10.1007/s10237-010-0274-y 21136134

[CR4] Cho HM, Choi SM, Park JY, Lee Y, Bae JH (2022) A finite element analysis and cyclic load experiment on an additional transcortical-type hole formed around the proximal femoral nail system’s distal locking screw. BMC Musculoskelet Disord 23:92. 10.1186/s12891-022-05049-0 35086522 PMC8793818

[CR5] Frank M, Fischer JT, Thurner PJ (2021) Microdamage formation in individual bovine trabeculae during fatigue testing. J Biomech 115:110131. 10.1016/j.jbiomech.2020.110131 33257009

[CR6] Garzón-Alvarado DA, Linero D (2012) Comparative analysis of numerical integration schemes of density equation for a computational model of bone remodelling. Comput Methods Biomech Biomed Eng 15(11):1189–1196 10.1080/10255842.2011.585972 21806414

[CR7] Garzón-Alvarado DA, Duque-Daza CA, Vaca-González JJ, Boucetta A, Linero DL, de Boer G, Das R, Ramtani S (2024) Part II: a new perspective for modeling the bone remodeling process: biology, mechanics, and pathologies. J Theor Biol 593:111894. 10.1016/j.jtbi.2024.111894 38992463

[CR8] Graham JM, Ayati BP, Ramakrishnan PS, Martin JA (2012) Towards a new spatial representation of bone remodeling. Math Biosci Eng 9(2):281–295. 10.3934/mbe.2012.9.281 22901065 PMC3708700

[CR9] Gray RJ, Korbacher GK (1974) Compressive fatigue behaviour of bovine compact bone. J Biomech 7(3):287–292. 10.1016/0021-9290(74)90020-7 4846499

[CR10] Günther T, Schinke T (2000) Mouse genetics have uncovered new paradigms in bone biology. Trends Endocrinol Metab 11(5):189–193 10856921 10.1016/s1043-2760(00)00256-3

[CR11] Hambli R (2014) Connecting mechanics and bone cell activities in the bone remodeling process: an integrated finite element modeling. Front Bioeng Biotechnol 2:6. 10.3389/fbioe.2014.00006 25152881 PMC4126454

[CR12] King AT, Evans FG (1967) Analysis of fatigue strength of human compact bone by the weibull method. In B. Jacobson, editor, Digest of the 7th International Conference on Medical and Biological Engineering, pages Stockholm, Royal Academy of Engineering Sciences,

[CR13] Kolken HMA, Garcia AF, Plessis AD, Meynen A, Rans C, Scheys L, Mirzaali MJ, Zadpoor AA (2022) Mechanisms of fatigue crack initiation and propagation in auxetic meta-biomaterials. Acta Biomater 138:398–409. 10.1016/j.actbio.2021.11.002 34763109

[CR14] Komarova SV, Smith RJ, Dixon SJ, Sims SM, Wahl LM (2003) Mathematical model predicts a critical role for osteoclast autocrine regulation in the control of bone remodeling. Bone 33(2):206–215 14499354 10.1016/s8756-3282(03)00157-1

[CR15] Lafferty JF, Raju PVV (1979) The influence of stress frequency on the fatigue strength of cortical bone. J Biomech Eng 101:112–113

[CR16] Lim C, Roh YH, Yoo SJ, Jeong DK, Nam KW (2022) Identification of stem cell related gene expression from the osteosarcoma cell core side. J Cancer Prev 27(2):122–128. 10.15430/JCP.2022.27.2.122 35864855 PMC9271406

[CR17] Lin X, Zhao J, Gao L, Zhang C, Gao H (2020) Ratcheting-fatigue behavior of trabecular bone under cyclic tensile-compressive loading. J Mech Behav Biomed Mater 112:104003. 10.1016/j.jmbbm.2020.104003 32823002

[CR18] Malekipour F, Hitchens PL, Whitton RC, Lee P-S (2022) Effects of in vivo fatigue-induced microdamage on local subchondral bone strains. J Mech Behav Biomed Mater 136:105491. 10.1016/j.jmbbm.2022.105491 36198232

[CR19] Martin B (1992) A theory of fatigue damage accumulation and repair in cortical bone. J Orthop Res 10(6):818–825. 10.1002/jor.1100100611 1403296

[CR20] Martin TJ, Ng KW (1994) Mechanisms by which cells of the osteoblast lineage control osteoclast formation and activity. J Cell Biochem 56(3):357–366 7876329 10.1002/jcb.240560312

[CR21] Meng X, Qu C, Fu D, Qu C (2021) Effects of fatigue damage on the microscopic modulus of cortical bone using nanoindentation. Materials 14(12):3252. 10.3390/ma14123252 34204688 PMC8231503

[CR22] Meng X, Qin Q, Qu C, Kang K, Wang Z, Qiu W, Qu C, Fu D (2023) The characterization of bovine compact bone fatigue damage using terahertz spectroscopy. Z Med Phys 33(2):192–202. 10.1016/j.zemedi.2022.06.001 35764468 PMC10311269

[CR23] Mouss ME, Zellagui S, Nasraoui M, Hambli R (2020) Parametric investigation of the effects of load level on fatigue crack growth in trabecular bone based on artificial neural network computation. Proceedings of the Institution of Mechanical Engineers, Part H: Journal of Engineering in Medicine 234(8):784–793. 10.1177/0954411920924509 32436783

[CR24] Oftadeh R, Perez-Viloria M, Villa-Camacho JC, Vaziri A, Nazarian A (2015) Biomechanics and mechanobiology of trabecular bone: a review. J Biomech Eng 137(1):0108021. 10.1115/1.4029176 25412137 PMC5101038

[CR25] Parfitt AM (1994) Osteonal and hemi-osteonal remodeling: the spatial and temporal framework for signal traffic in adult human bone. J Cell Biochem 55(3):273–286 7962158 10.1002/jcb.240550303

[CR26] Pouca MCPV, Parente MPL, Jorge RMN, Ashton-Miller JA (2020) Investigating the birth-related caudal maternal pelvic floor muscle injury: the consequences of low cycle fatigue damage. J Mech Behav Biomed Mater 110:103956. 10.1016/j.jmbbm.2020.103956 32957249 PMC9016363

[CR27] Brand RA (2010) Biographical sketch: Julius Wolff, 1836–1902. Clin Orthop Relat Res 1(10):717–726. 10.1007/s11999-010-1258-z PMC2835589 20151232

[CR28] Ramtani S, Sánchez JF, Boucetta A, Kraft R, Vaca-González JJ, Garzón-Alvarado DA (2023) A coupled mathematical model between bone remodeling and tumors: a study of different scenarios using komarova’s model. Biomech Model Mechanobiol 22(3):925–945. 10.1007/s10237-023-01689-3 36922421 PMC10167202

[CR29] Ramtani S, Sánchez JF, Boucetta A, Kraft R, Vaca-González JJ, Garzón-Alvarado DA (2023) A coupled mathematical model between bone remodeling and tumors: a study of different scenarios using komarova’s model. Biomech Model Mechanobiol. 10.1007/s10237-023-01689-3 PMC10167202 36922421

[CR30] Ramtani S, Toudji L, Boucetta A, Boukharouba T, Garzón-Alvarado DA (2023) Komarova’s bone remodeling type model revisited within the context of a new parameter affecting both production and removal activities of osteoblasts and osteoclasts. Journal of Mechanics in Medicine and Biology

[CR31] Sánchez JF, Ramtani S, Boucetta A, Velasco MA, Vaca-González JJ, Duque-Daza CA, Garzón-Alvarado DA (2024) Tumor growth for remodeling process: a 2d approach. J Theor Biol 585:111781. 10.1016/j.jtbi.2024.111781 38432504

[CR32] Santalla A, Valenzuela PL, Rodriguez-Lopez C, Rodríguez-Gómez I, Nogales-Gadea G, Pinós T, Arenas J, Martín MA, Santos-Lozano A, Morán M, Fiuza-Luces C, Ara I, Lucia A (2022) Long-term exercise intervention in patients with mcardle disease: clinical and aerobic fitness benefits. Med Sci Sports Exerc 54(8):1231–1241. 10.1249/MSS.0000000000002915 35320153

[CR33] Shigley JE, Budynas RG (2019) Diseño en ingeniería mecánica de shigley. McGraw Hill Education, 10th edition. ISBN 978-1456267568

[CR34] Tyrovola JB (2015) The “mechanostat theory’’ of frost and the opg/rankl/rank system. J Cell Biochem 116(12):2724–9 26096594 10.1002/jcb.25265

[CR35] Von Meyer G (1867) The architecture of the spongy substance and the formation of the cancellous bone tissue. F.C.W Vogel, Leipzig

[CR36] Whalen RT, Carter DR, Steele CR (1988) Influence of physical activity on the regulation of bone density. J Biomech 21(10):825–837. 10.1016/0021-9290(88)90015-2 3225269

[CR37] Wolff J (1873) Zur lehre von der fracturenheilung. Langenbecks Arch Klin Chir Ver Dtsch Z Chir 2:546–551

